# Discovery of Novel Ligands for Mouse Olfactory Receptor MOR42-3 Using an *In Silico* Screening Approach and *In Vitro* Validation

**DOI:** 10.1371/journal.pone.0092064

**Published:** 2014-03-17

**Authors:** Selvan Bavan, Benjamin Sherman, Charles W. Luetje, Tatjana Abaffy

**Affiliations:** Department of Molecular and Cellular Pharmacology, Miller School of Medicine, University of Miami, Miami, Florida, United States of America; Monell Chemical Senses Center, United States of America

## Abstract

The ligands for many olfactory receptors remain largely unknown despite successful heterologous expression of these receptors. Understanding the molecular receptive range of olfactory receptors and deciphering the olfactory recognition code are hampered by the huge number of odorants and large number of olfactory receptors, as well as the complexity of their combinatorial coding. Here, we present an *in silico* screening approach to find additional ligands for a mouse olfactory receptor that allows improved definition of its molecular receptive range. A virtual library of 574 odorants was screened against a mouse olfactory receptor MOR42-3. We selected the top 20 candidate ligands using two different scoring functions. These 40 odorant candidate ligands were then tested *in vitro* using the *Xenopus* oocyte heterologous expression system and two-electrode voltage clamp electrophysiology. We experimentally confirmed 22 of these ligands. The candidate ligands were screened for both agonist and antagonist activity. In summary, we validated 19 agonists and 3 antagonists. Two of the newly identified antagonists were of low potency. Several previously known ligands (mono- and dicarboxylic acids) are also confirmed in this study. However, some of the newly identified ligands were structurally dissimilar compounds with various functional groups belonging to aldehydes, phenyls, alkenes, esters and ethers. The high positive predictive value of our *in silico* approach is promising. We believe that this approach can be used for initial deorphanization of olfactory receptors as well as for future comprehensive studies of molecular receptive range of olfactory receptors.

## Introduction

The olfactory receptor gene family is the largest gene family in the mammalian genome [1], [2]. There are approximately 1035 mouse olfactory receptors. Based on the phylogenetic analysis these receptors are categorized in 228 families, each sharing more than 40% sequence identity [3]. Olfactory receptor family detects and distinguishes a huge number of odorants in a combinatorial fashion, meaning that one odorant can be recognized by many different receptors and that one receptor can recognize multiple odorant structures [4]. In order to study chemical recognition and olfactory coding, we need to deorphanize olfactory receptors and define their molecular receptive ranges. Despite the availability of heterologous expressions systems, most mammalian olfactory receptors are still waiting to be deorphanized [5], [6], [7]. Identifying olfactory receptor-ligand pairs is challenging for several reasons, including a) the large number of olfactory receptors that must be screened, b) the huge number of odorants, c) the heterogeneity in odorant structure and thus physicochemical properties, and d) the wide concentration range at which odorants may be active. So far, approximately 100 mouse olfactory receptors have been deorphanized [5], [6], [8], [9], [10], [11], [12], [13], [14]. In the largest study so far, 52 out of 219 mouse olfactory receptors (23%) screened *in vitro* by Saito et al, were deorphanized using a selected set of 93 odorants [6]. The full molecular receptive ranges of these receptors, however, have yet to be investigated.

In order to measure odorant similarity/dissimilarity and to visualize odorant position within in the huge odor space, Haddad et al. generated a multidimensional odor-map, where initially each odorant was represented by >1,000 molecular descriptors which were optimized to the 32 most salient descriptors [15]. Similarly, Saito et al. analyzed the correlation between receptor responses and various molecular descriptors from a set of 93 odorants [6] and found that 18 molecular descriptors are able to explain >62% of the variance in the mouse and human olfactory receptor responses. Thus, analyzing molecular descriptors of various odorants and placing them on the odor map enables us to measure the odor space representative of a particular olfactory receptor and to evaluate whether a receptor is broadly or narrowly tuned [16], [17]. Still, the heterogeneity of odorants makes *in vitro* screening strategies particularly challenging and labor intensive.

Here we present another approach to study the molecular receptive range of olfactory receptors. We first applied virtual ligand screening to find additional ligands and to further characterize the molecular receptive range of MOR42-3. Next, we validated our *in silico* results with *in vitro* testing of top scoring compounds using the *Xenopus* oocyte heterologous expression system and functional assay by electrophysiology. MOR42-3 is a class I or “fish-like” olfactory receptor [3]. We previously showed that MOR42-3 responds primarily to 8-10 carbon linear dicarboxylic acids; with nonanedioic acid being the preferred ligand [5]. Here, we used a previously developed homology model of MOR42-3 [18] for docking a library of 574 odorants using Internal Coordinate Mechanics (ICM) software (MolSoft, LLC, La Jolla, CA). We employed two different scoring functions to estimate the strength of the receptor-ligand interaction, producing two lists of the top 20 candidate-binders. These 40 compounds were then tested *in vitro* for agonist, as well as for antagonist activity. From the first list (based on score function) we identified 10 agonists and 1 antagonist and from the second list (based on mf score function) we identified 9 agonists and 2 antagonists. We believe that this approach can be used for initial deorphanization of olfactory receptors as well as for future comprehensive studies of molecular receptive ranges of olfactory receptors.

## Materials and Methods

### *In silico* assay

For virtual ligand screening (VLS), we used ICM 3.7-2-d modeling software on a 3.0 GHz Intel Xeon processor (MolSoft LLC, San Diego, CA) [19]. The MOR42-3 homology model was built based on rhodopsin crystal structure (1U19) as described previously [18]. The sequence alignment of MOR42-3 and rhodopsin with underlined transmembrane domains is presented in **Figure S1**. The pdb file of MOR42-3 model is presented in the **Figure S2**. Our homology model based on rhodopsin template 1U19 with crystallographic resolution of 2.2A has been functionally characterized and validated through mutagenesis experiments, which gave us extreme confidence in the accuracy of our model [18]. Based on our previous work, we selected ligand binding pocket residues using graphical tools in the ICM software. This information was used to create a box that defined the boundaries of the docking search. Potential energy maps of the receptor on a 0.5 Å grid were calculated using default parameters. For odorant library construction, 574 compounds were imported into an ICM molecular table, converted to 3D and optimized. An index file for all compounds was created. We used default docking preferences with the exception of flexible ring sampling level of 2.0 and docking thoroughness of 2.0. Flexible ring sampling level of 2.0 means that the ring in macrocycles (cyclic compounds with more than 9 atoms) is flexible throughout the docking simulation, as opposed to 0, which is default setting with no flexibility allowed. Docking thoroughness represents the length of the simulation and it is slightly increased as we have large pocket to search. Conformational sampling was based on the Monte Carlo procedure, which randomly selects a conformation in the internal coordinate space and makes a new random position independent of the previous one. From the complete VLS hit list presented in **Table S1**, two top 20 lists were generated, first by sorting compounds for best docking score (score ≤−24.49) and second by sorting compounds for best mfscore (mfscore ≤ −109.77). *The score in ICM software* is based on the empirical function of predicted physical interaction terms as described in the following formula:
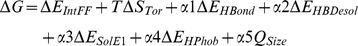



This predicted score (kcal/mol) is calculated as the weighted (α1–α5) sum of ligand-target van der Waals interactions and internal force field energy of the ligand (ΔE_IntFF_), free energy changes due to conformational energy loss upon ligand binding (TΔS_Tor_), hydrogen bonding interactions (ΔE_HBond_), hydrogen bond donor-acceptor desolvation energy (ΔE_HBDesol_), solvation electrostatic energy upon ligand binding (ΔE_SolE1_), hydrophobic free energy gain (ΔE_HPhob_)and a size correction term proportional to the number of ligand atoms (Q_Size_), as described in detail in the Neves et al. paper [20]. On the contrary, *mfscore in ICM software* is a potential of mean force score and provides an independent score of the strength of ligand-receptor interaction. It is a measure of statistical probability of interaction between the ligand and the receptor. It examines interatomic distances of the docked interaction, and compares that to existing interactions available in PDB [21]. The mean force Score (mfScore) is calculated following methodology of Muegge &Martin [22]. It is a knowledge-based potential derived from frequencies of occurrence of various atom pairs at different distances within experimental ligand/receptor complex structures in PDB. While different scoring functions are all correlated to binding energies, they are inevitably only approximations of the true values, with a mix of various systematic and/or random errors. Thus ligand ranking according to one scoring function can differ significantly from ranking according to another scoring function, especially when one is based on physical interaction term contributions while the other is a knowledge-based function. Consensus scoring that utilizes combination of scoring functions has been shown to improve hit rates as shown by Bissantz et al. [23] (personal communication with M. Totrov and A. Orry, MolSoft).

Top 20 compounds from each list (**Table 1** **and** **Table 2**) were then evaluated for their ability to act either as agonists or antagonists of MOR42-3 using *in vitro* heterologous expression and functional assay (see below). Fingerprint (FP) similarity distance was calculated in ICM Chemical search by MolSoft software, where 0.9999 was set as a max distance or dissimilarity.

**Table 1:** Properties of the top 20 candidates as ranked by VLS score.

Rank	Name	Formula	Mol Weight	Score	Mol LogP	Mol Volume	mfScore
1	glutaric acid	C_5_H_8_O_4_	132.04	−42.01	−0.05	121.52	−11.49
2	succinic acid	C_4_H_6_O_4_	118.03	−40.81	−0.53	103.62	−25.76
3	adipic acid	C_6_H_10_O_4_	146.06	−40.15	0.43	139.43	−20.05
4	malonic acid	C_3_H_4_O_4_	104.01	−36.55	−0.96	85.29	−18.27
5	heptanedioic acid	C_7_H_12_O_4_	160.07	−35.61	0.92	157.34	−4.98
**6**	**9-methoxy-9-oxo-nonanoic acid**	**C_10_H_18_O_4_**	**202.12**	**−32.99**	**2.23**	**216.15**	**−76.98**
**7**	**8-methoxy-8-oxo-octanoic acid**	**C_9_H_16_O_4_**	**188.10**	**−30.20**	**1.75**	**198.25**	**−98.29**
8	oxalic acid	C_2_H_2_O_4_	90.00	−29.72	−0.54	67.91	−30.76
**9**	**undec-2-enal**	**C_11_H_20_O**	**168.15**	**−28.66**	**4.35**	**215.74**	**−103.73**
**10**	**undecanedioic acid**	**C_11_H_20_O_4_**	**216.14**	**−27.67**	**2.84**	**228.96**	**−104.30**
**11**	**nonanedioic acid**	**C_9_H_16_O_4_**	**188.10**	**−27.50**	**1.88**	**193.15**	**−97.71**
**12**	**nonanedioyl chloride**	**C_9_H_14_Cl_2_O_2_**	**224.04**	**−26.89**	**3.41**	**215.39**	**−84.05**
*13*	*dodecanedioic acid*	*C_12_H_22_O_4_*	*230.15*	−*26.78*	*3.33*	*246.87*	−*105.11*
**14**	**octanedioyl chloride**	**C_8_H_12_Cl_2_O_2_**	**210.02**	**−26.75**	**2.93**	**197.48**	**−88.72**
15	4-(7,9-dioxa-bicyclo[4.3.0]nona-1,3,5-trien-3-yl)-butan-2-one	C_11_H_12_O_3_	192.08	−26.00	2.44	198.90	−89.87
**16**	**octanedioic acid**	**C_8_H_14_O_4_**	**174.09**	**−25.29**	**1.40**	**175.24**	**−10.78**
**17**	**alpha-hexyl cinnamaldehyde**	**C_15_H_22_O**	**216.15**	**−25.24**	**4.98**	**255.25**	**−53.42**
**18**	**3-(4-(2-carboxy-ethyl)-phenyl)-propanoic acid**	**C_12_H_14_O_4_**	**222.09**	**−24.98**	**1.81**	**214.27**	**−102.90**
19	1,4-diethyl butanedioate	C_8_H_14_O_4_	174.09	−24.57	1.12	183.64	−41.03
20	butyric acid	C_4_H_8_O_2_	88.05	−24.49	0.87	91.05	−5.17

Compounds 6, 7, 9, 10, 11, 12, 14, 16, 17 and 18, confirmed as agonists in *in vitro* assay, are presented in bold. Compound 12, dodecanedioic acid, confirmed as antagonist, is presented in italics.

**Table 2:** Properties of the top 20 candidates as ranked by mfscore.

Rank	Name	Formula	Mol Weight	Score	Mol LogP	Mol Volume	mfScore
1	(3R,7S,11S)-3,7,11,15-tetramethyl-hexadec-1-en-3-ol	C20H40O	296.30	−17.84	8.2	380.16	−134.15
2	1-methyl-ethyl 2-phenyl-ethanoate	C11H14O2	178.09	−16.88	2.49	182.66	−127.64
*3*	*2-phenyl-ethyl 2-phenyl-ethanoate*	*C16H16O2*	*240.11*	−*15.43*	*3.68*	*239.78*	−*127.33*
4	oleic acid	C18H34O2	283.26	−13.7	7.15	348.59	−125.83
5	pentyl hexanoate	C11H22O2	186.16	−17.26	4.11	221.09	−123.2
*6*	*(2S,3S)-ethyl 3-methyl-3-phenyl-oxirane-2-carboxylate*	*C12H14O3*	*206.09*	−*16.69*	*2.59*	*221.12*	−*121.56*
7	5-methyl-2-phenyl-hex-2-enal	C13H16O	188.1	−22.18	3.71	216.98	−121.47
**8**	**dodecanal**	**C12H24O**	**184.18**	**−19.39**	**4.83**	**230.08**	**−120.81**
**9**	**decanoic acid**	**C10H20O2**	**173.15**	**−7.26**	**3.76**	**198.49**	**−118.83**
**10**	**pentyl pentanoate**	**C10H20O2**	**172.14**	**−15.25**	**3.63**	**203.18**	**−118.7**
11	undecanal	C11H22O	170.16	−16.06	4.35	212.17	−118.36
**12**	**undec-10-enoic acid**	**C11H20O2**	**185.15**	**−10.29**	**3.86**	**220.24**	**−117.71**
**13**	**4-(4-hydroxy-4-methyl-pentyl)-cyclohex-1-enecarbaldehyde**	**C13H22O2**	**210.16**	**−15.9**	**3.69**	**268.82**	**−117.04**
14	berberine chloride	C20H18ClNO4	371.81	−15.64	5.38	340.03	−115.84
**15**	**allyl heptanoate**	**C10H18O2**	**170.13**	**−19.94**	**3.41**	**206.86**	**−115.77**
**16**	**allyl 2-phenyl-ethanoate**	**C11H12O2**	**176.08**	**−20.08**	**2.5**	**189.42**	**−114.24**
**17**	**2-phenyl-ethyl hexanoate**	**C14H20O2**	**220.14**	**−16.46**	**4.11**	**239.31**	**−112.76**
**18**	**undecanoic acid**	**C11H22O2**	**187.17**	**−6.89**	**4.24**	**216.39**	**−111.19**
19	nonanoic acid	C9H18O2	158.13	−24.19	3.28	180.58	−110.39
20	tetradecanoic acid	C14H28O2	229.21	1.74	5.69	270.11	−109.77

Compounds 8, 9, 10, 12, 13, 15, 16, 17 and 18, confirmed as agonists in *in vitro* assay are presented in bold. Compounds 3 and 6 confirmed as antagonists are presented in italics.

### In vitro assay

Ethics statement: Xenopus laevis frogs used in this study were purchased from Nasco (Fort Atkinson, Wi, USA). Female X. laevis frogs (2–4 years old) were housed in an AAALAC accredited aquatic animal facility. This study was carried out in strict accordance with the recommendations in the Guide for the Care and Use of Laboratory Animals of the National Institutes of Health. The protocol was approved by the University of Miami Institutional Animal Care and Use Committee. For oocyte isolation, frogs were anesthetized by submersion in 0.1% 3-aminobenzoic acid ethyl ester. Oocytes were then surgically removed and the incision treated with gentamicin and sutured. All efforts were made to minimize suffering. Frogs were allowed to recover from surgery in a humid chamber, before being placed in an isolation tank for 24 hours, and then returned to the general population. The surgically obtained oocytes were treated with collagenase B (Boehringer - Mannheim) for 2 hours at room temperature to remove follicle cells.

Oocytes were maintained at 18 °C in Barth's saline solution (in mM: 88 NaCl, 1 KCl, 2.4 NaHCO3, 0.3 CaNO3, 0.41 CaCl2, 0.82 MgSO4, 15 HEPES, pH 7.6 and 12 μg/mL tetracycline). Expression constructs for MOR42-3, Gαolf and the Cystic Fibrosis Transmembrane Regulator (CFTR) were generated as previously described (Abaffy et al., 2006). Capped cRNAs encoding MOR42-3, Gαolf and CFTR were transcribed using mMESSAGE MACHINE kits (Ambion). Stage V oocytes were injected with 46 μL water containing 40 ng of MOR42-3 cRNA, 10 ng Gαolf cRNA and 1.5 ng CFTR cRNA. Oocytes were incubated at 18 °C in Barth's saline solution for 2-5 days prior to electrophysiological recordings. Odorants were stored under argon and high concentration stock solutions of each odorant were prepared in dimethyl sulfoxide (DMSO) except for citric acid (CAS: 77-92-9) and berberine chloride (CAS: 633-65-8) which were dissolved straight into ND96, and N, N-dimethyl-methanamine (CAS: 5-50-3) which was dissolved in ethanol. These stocks were subsequently diluted in ND96 to obtain 100 μM (up to 0.1% DMSO) odorant concentration for agonist assays and 1 mM (up to 0.5% DMSO) for antagonist assays.

Two-electrode voltage clamp in an automated parallel electrophysiology system (OpusExpress 6000A, Molecular Devices, Union City, CA, USA) was used to measure odorant-induced Cl^−^ currents as previously described (Abaffy et al., 2006). Micropipettes were filed with 3 M KCl, with resistances of 0.2–2.0 MΩ. The holding potential was −70 mV. Current responses, filtered (4-pole, Bessel, low pass, at 20 Hz 3db) and sampled at 100 Hz, were captured and stored using OPUSXPRESS 1.1 software (Molecular Devices). Initial analysis was performed using CLAMPFIT 9.1 software (Molecular Devices). Oocytes were perfused under ND96 (in mM: 96 NaCl, 2 KCl, 1CaCl2, 1 MgCl2, 5 HEPES, pH 7.4). All odorants were applied for 15 seconds. 1 mM 3-isobutyl-1-methylxanthine (IBMX) was used to demonstrate the ability of the oocyte to functionally express the injected cRNA. For quantitation of odorant-evoked responses in agonist assays, test odorant-responses were normalized to the average response of two 100 μM nonanedioic acid applications (applied 20 minutes before and 20 minutes after test odorant application). For quantitation of antagonist activity, the test odorant at 1 mM was co-applied with 10 μM nonanedioic acid, and the response was normalized to the average of two 10 μM nonanedioic acid applications (one application 20 minutes before and 20 minutes after test odorant application). Our previous results [5], [18] indicate that for MOR42-3, nonanedioic acid is the most potent agonist with the EC50 in the low micromolar range (5.9 ± 0.9 μM). We choose to test our potential agonists at the 100 μM concentration to ensure maximal potency. Antagonism was assessed at 1 mM to allow identification of even low potency compounds. Data are expressed as mean ± SEM. Odorant responses were not analyzed when nonanedioic acid responses were less than 50 nA. The bottom 20 compounds from the VLS list sorted by score **(Table S1)** were also tested using mixtures composed of 5 odorants. None of the compounds activated MOR42-3 (results not shown).

## Results

**Figure 1:**
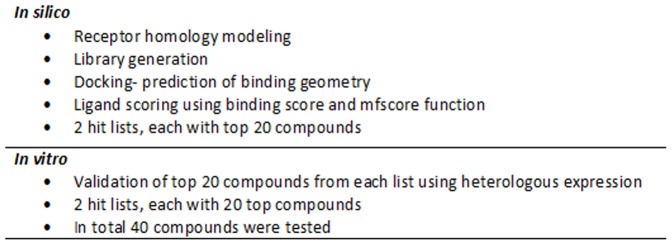
The experimental study design. List of procedures used in this study for *in silico* and *in vitro* approach of deorphanizing olfactory receptors.

In order to find additional ligands for mouse olfactory receptor MOR42-3, we used an in silico, structure based virtual ligand screening (VLS) approach. Our experimental study design is presented in Figure 1. In our previous work, homology modeling of MOR42-3 receptor and docking analysis of various dicarboxylic acids predicted a network of ligand binding residues for this particular receptor. We were able to show that by mutating these residues we could change receptor ligand preference. In particular we were able to the change ligand preference of MOR42-3 to the ligand preference of MOR42-1. Thus, we were able to experimentally confirm our *in silico* prediction [18]. In the present study, a diverse panel of 574 odorant compounds was screened against this validated homology model of MOR42-3.

VLS using ICM docks flexible compounds into a grid map of a receptor structure and evaluates each docked conformation with a scoring function. These ICM scores are calculated from hydrophobicity, solvation electrostatics, hydrogen bonding, ligand deformation energy and van der Waals ligand-receptor interaction energy [20]. In general, this scoring function is used to discriminate binders from non-binders, with more negative scores representing more likely binding interactions and higher binding affinity of a particular ligand. The docking scores for 574 compounds in our odorant panel showed a normal distribution (**Figure S3**) and a score ranked list is presented in **Table S1**. This table also includes several other molecular descriptor parameters for each potential ligand. Our rationale for the choice of the score thresholds was set arbitrarily to the best 20 compounds. The 20 best scoring compounds (**Table 1**) had scores ranging from −42 to −24.5. In addition to this ICM binding score, we also ranked the docking results based on the mfScore function, which is based on a potential of mean force calculation and provides an independent score of the strength of ligand-receptor interaction [19]. The 20 best scoring compounds according to this criterion, with mfScores ranging from −134 to −110, are presented in **Table 2**.

**Figure 2:**
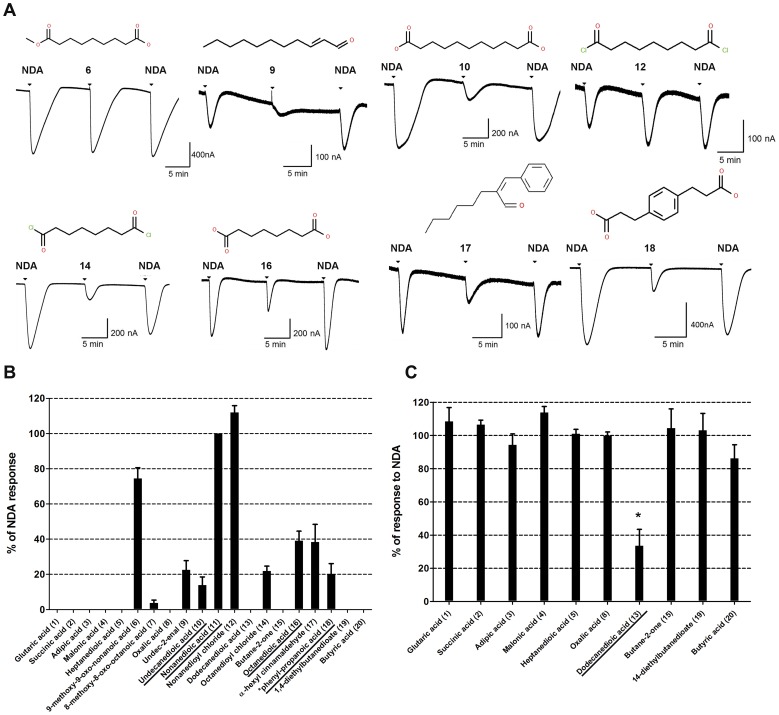
*In vitro* validation of *in silico* results. **A.** Representative traces of agonists, their structures and corresponding screen numbers tested at 100 μM for 15sec. Responses to 100 μM nononedioic acid (NDA) before and after application of each odorant was used for normalization. **B.** Histogram showing relative activity of the top 20 candidate ligands listed according to their score, tested at 100 μM and normalized to nonanedioic acid (mean±SEM, n = 5–10). Compound 18 full name is * 3-(4-(2-carboxyethyl)-phenyl)-propanoic acid. **C.** Histogram showing responses of ligands tested as antagonists at 1mM concentration and in the presence of 10 μM NDA. Responses are expressed as % of the responses to 100 μM nonanedioic acid, mean±SEM, n = 5–10. Compound 13 (dodecanoic acid) is an antagonist, *p<0.05. This compound (underlined) was previously identified as an antagonist for MOR42-3 [18].

**Figure 3:**
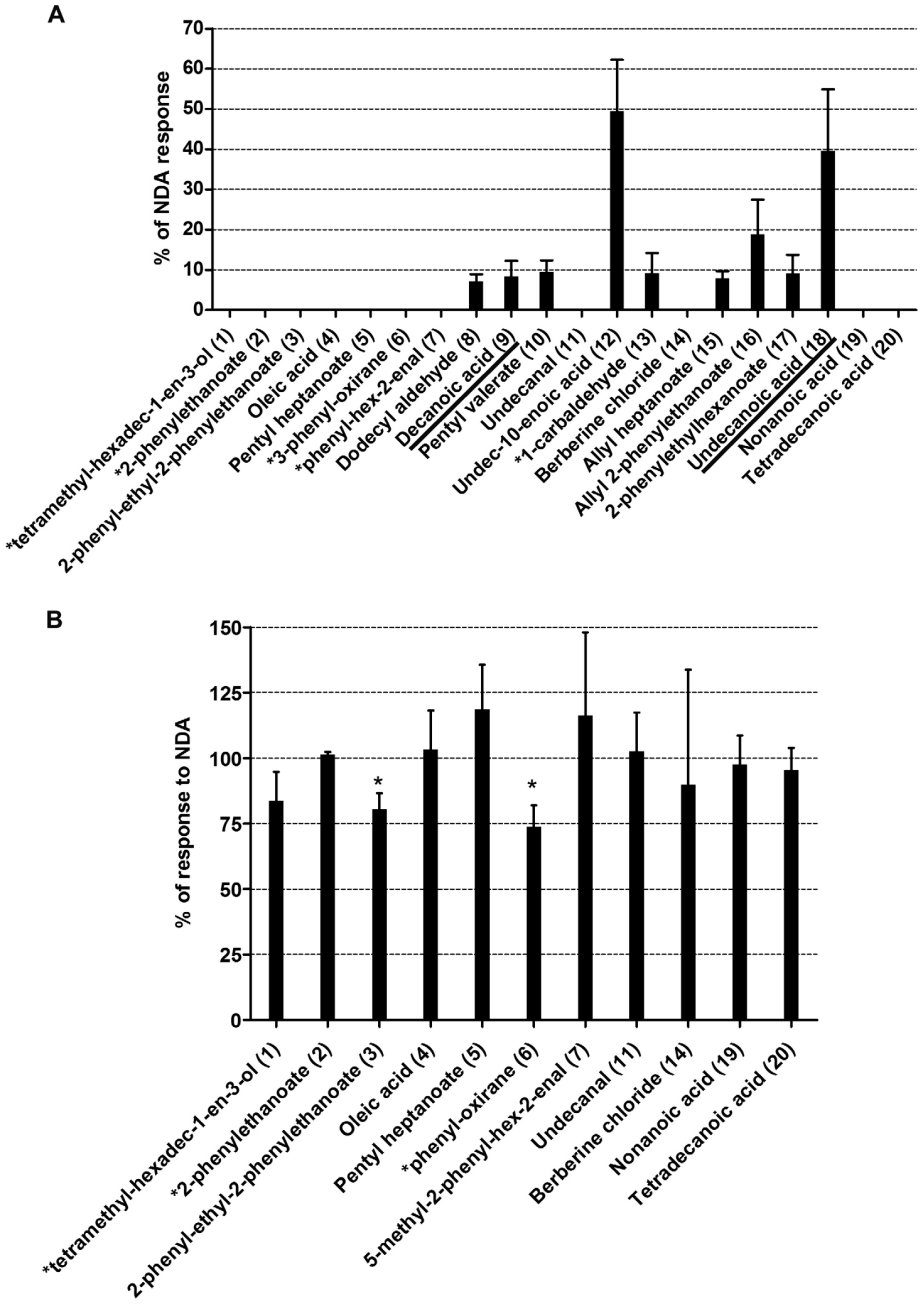
*In vitro* validation of *in silico* results. **A.** Histogram showing relative activity of the top 20 candidate ligands listed according to their mfscore, tested at 100 μM and normalized to the nonanedioic acid, mean±SEM, n = 5–10. Full name of the compounds: Compound 1 = 3,7,11,15-tetramethyl-hexadec-1-en-3-ol (1); Compound 2 = 1-methyl-ethyl-2-phenylethanoate (2); Compound 6 =  ethyl-3-methyl-3-phenyl-oxirane-2-carboylate (6); compound 7 = 5-methyl-2-phenyl-hex-2-enal (7) and compound 13 = 4-(4-hydroxy-4-methylpentyl)-cyclohexene-1-carbaldehyde (13). **B.** Histogram showing response of ligands tested as antagonists at 1mM concentration, in the presence of 10 μM NDA. Compounds 3 and 6 were identified as low potency antagonists, *p<0.0025. Previously identified ligands for MOR42-3 confirmed in our *in silico* screen are underlined.

For *in vitro* validation of our *in silico* results we expressed MOR42-3 in *Xenopus* oocytes and assayed receptor function using two-electrode voltage clamp electrophysiology (see Materials and Methods). We tested the 20 best scoring ligands from each ranking list (**Tables 1** **and** **2**) for agonist activity at 100 μM. Current responses were normalized to the mean response to nonanedioic acid (a known agonist of this receptor) and are presented in **Figures 2** and **3**. Several previously known agonists for MOR42-3 (underlined in **Figures 2B** and **3A**) were identified in the screen, either by using the score or mfscore functions, supporting the validity of our *in silico* screening approach. In addition, we identified several novel agonists for this receptor. However, this screen did not identify all known agonists of MOR42-3. These compounds were tested in an *in vitro* assay and confirmed to activate MOR42-3. Previously identified agonist 5-oxonononanedioic acid [5] was not included in the initial screen of 574 compounds and thus was not tested. Our results from *in vitro* assay, show that both undecanal and nonanoic acid (compounds 11 and 19 from **Table 2**) are able to activate receptor at 1 mM concentration, but not at 100 μM (with a mean and SEM of 134.5 ± 34.8, n = 7 and 89.9 ± 43.8, n  =  8, respectively, results not shown). Decanedioic and dodecanoic acid, previously identified as MOR42-3 agonists were not ranked in either top 20 list; decanoic acid was ranked at 367 in the VLS, with a score of −14.47 and an mfScore of −62.40 and dodecanoic acid ranked at 499 in VLS with score of −10.45 and an mfScore of −79.91 (see **Table S1**).

Compounds that failed to activate MOR42-3 were then tested for the ability to antagonize the response of MOR42-3 to nonanedioic acid (**Figures 2C** and **3B**). As expected from our previously published work [18], dodecanedioic acid (present in the top 20 list from the score function) antagonized nonanedioic acid activation of the receptor (**Figure 2C**). While this compound was tested here at 1 mM against μM agonist (nonanedioic acid), we previously showed that dodecanedioic acid can also exert antagonist activity at a lower concentration (100 μM) when tested against a higher nonanedioic acid concentration (100 μM). None of the other non-agonist compounds from the score function list displayed any antagonist activity. We also observed little or no antagonist activity with the non-agonist compounds from the mfscore list. Two compounds (2-phenyl-ethyl 2-phenyl-ethanoate, No. 3 and (2S, 3S)-ethyl 3-methyl-3-phenyl-oxirane-2-carboxylate, No 6 from **Table 2**) displayed modest antagonist activity when applied at the relatively high concentration of 1 mM against 10 μM nonanedioic acid. When we tested 2-phenyl-ethyl 2-phenyl-ethanoate under more challenging conditions (100 μM compound versus 100 μM nonanedioic acid), no significant antagonism was observed (95 ± 9% of control, mean ± SEM), indicating that this compound is of very low potency. From the top 20 compounds based on the docking score values, we identified 10 agonists and 1 antagonist, while from the top 20 compounds based on mfscore values, we identified 9 agonists and 2 antagonists. While several of the agonists displayed only modest activity (as compared to nonanedioic acid) and two of the antagonists are low potency, we are including compounds with any agonist or antagonist activity in the following analyses. We will then be more selective when considering some of the novel agonist compounds. Our results indicate that that both scoring functions have a similar positive predictive value (PPV) of 55% (PPV_s_ = 10+1/20 and PPV_mfs_ = 9+2/20, respectively) and that both can be used for future screening and evaluation studies. Several of the compounds identified in our *in silico* screen, using the score or mfscore function, have been previously identified as ligands for MOR42-3 (underlined in **Figure 2** and **Figure 3**). As a further test of the validity of our screening strategy, the 20 worst scoring compounds from our VLS screen, with scores ranging from -4.88 to 20.17, were also tested for agonist and antagonist activity against MOR42-3 expressed in *Xenopus* oocytes (the last 20 compounds listed in **Table S1**). None of these compounds showed any agonist or antagonist activity (data not shown).

**Figure 4:**
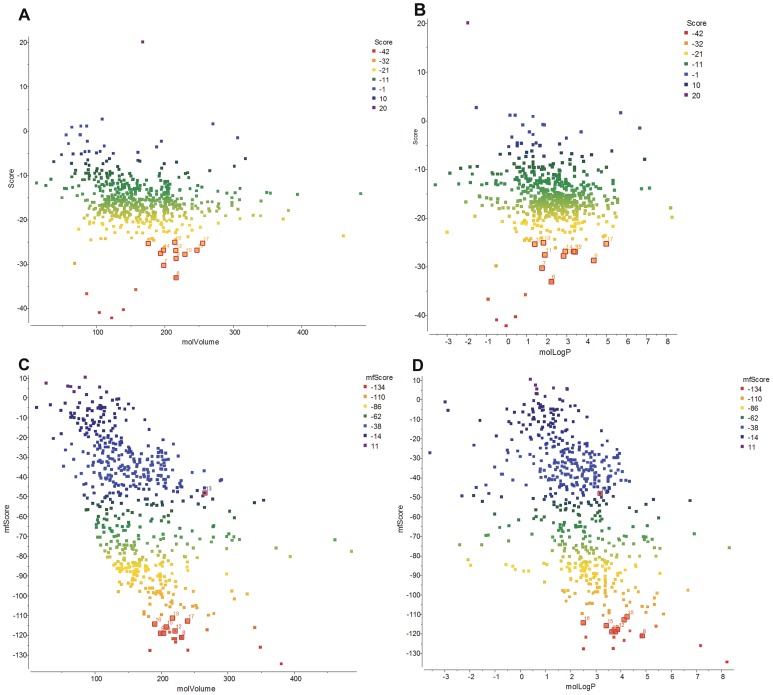
Clustering of the ligands based on their molecular properties. **A.** VLS dot plot of score values *vs* molecular volume. Compounds acting as ligands from the **Table 1** are labeled. Color scheme represents scoring as indicated. **B.** VLS dot plot of score values *vs* log P for compounds from **Table 1** are labeled. **C.** VLS dot plot of mfscore values *vs* molecular volume. Compounds acting as ligands from the **Table 2** are labeled. **D.** VLS dot plot of score values *vs* log P for compounds from **Table 2** are labeled.

Agonists and antagonists for MOR42-3 that were confirmed in the *in vitro* assay had molecular volumes ranging from 175 to 255 Å^3^ (see bold and italicized in **Table 1** and **Table 2**). VLS dot plot results showing a relationship between score or mfscore values and molecular volume (**Figure 4A and 4C**) indicate that ligands for this receptor cluster together within a small range of molecular volume. Most of the top 20 *in silico* predicted compounds that did not activate or inhibit the MOR42-3 receptor in the *in vitro* assay (compounds 1,2,3,4,5,8,19 and 20) had smaller molecular volumes ranging from 67-183 Å^3^. These compounds also showed docking conformations higher up in the receptor cavity, as compared to the *in vitro* validated compounds (data not shown). Log P, an octanol/water partitioning ratio of an unionized compound, is a measure of hydrophobicity. **Figures 4B and 4D** show strong clustering of MOR42-3 ligands based on their log P value and the two scoring functions. These results indicate that, in addition to molecular volume, hydrophobicity is an important characteristic for ligands that successfully interact with the MOR42-3 receptor.

**Figure 5:**
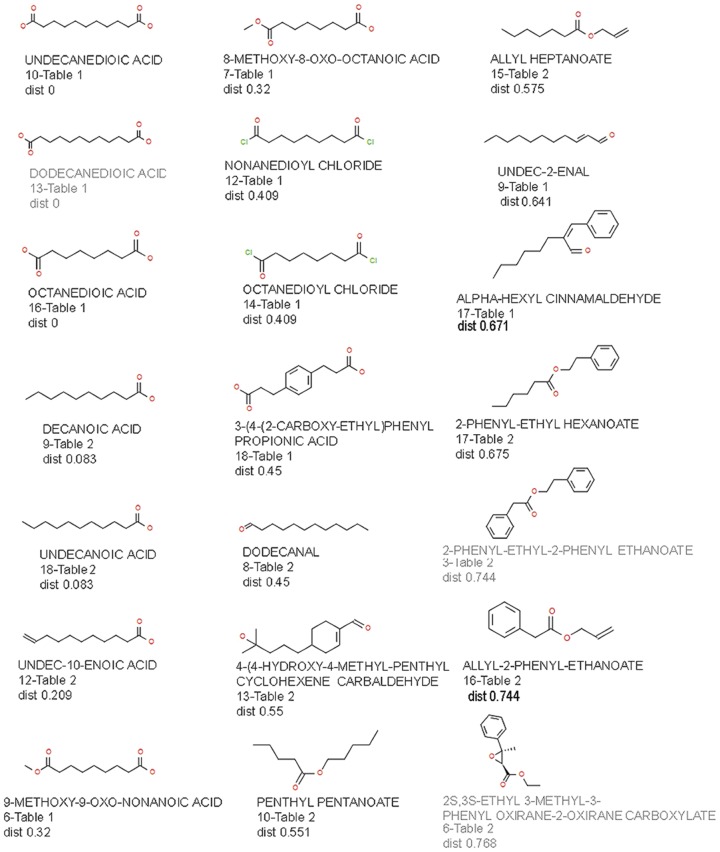
Ligands for MOR42-3 identified by VLS screening and their structural similarity to nonanedioic acid expressed as a fingerprint distance from nonanedioic acid. (Antagonists are labeled in grey).

**Figure 6:**
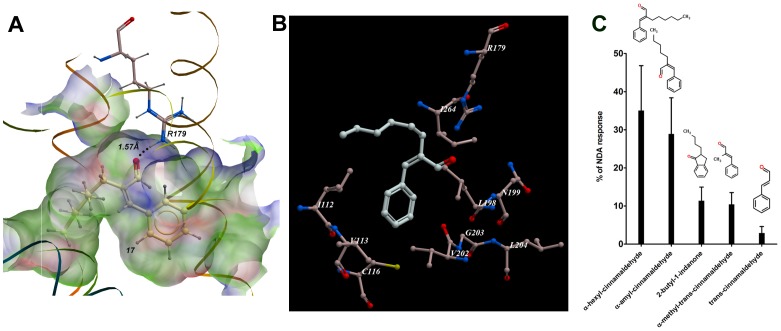
Agonist binding pocket and additional ligands for MOR42-3. **A.** α-hexyl cinnamaldehyde docked in MOR42-3. Ligand binding pocket is colored by binding property with green representing hydrophobic areas, red hydrogen bond acceptors and blue hydrogen bond donors. Hydrogen bond between carbonyl oxygen from α-hexyl cinnamaldehyde (ranked 17, **Table 1**) and guanidinuim group of arginine 179 (R179) is presented with an interatomic distance of 1.57Å. **B.** Residues within 5Å distance from the best docking conformation of α-hexyl cinnamaldehyde. **C.** Structural relatives of α-hexyl cinnamaldehyde and their responses. Oocytes expressing MOR42-3, Gαolf and CFTR were screened with 15 sec application of 100 μM indicated odorants. Responses were normalized to the average 100 μM NDA-evoked responses and results are presented as a mean±SEM, n =  5–10.

Both linear and cyclic compounds were identified and validated as ligands for MOR42-3. These compounds were diverse, encompassing aldehydes, phenyls, alkenes, esters, ethers, and monocarboxylic and dicarboxylic acids. The structure of each validated compound, VLS ranking and fingerprint similarity distance from nonanedioc acid is presented in **Figure 5**. By using a value of 0.5 as a nonsimilarity threshold, we found that 9 ligands (7 agonists and 2 antagonists) are not similar to nonanedioic acid. Nevertheless, these compounds can act as agonists (compounds 9 and 17 from **Table 1** and compounds 10, 13, 15, 16 and 17 from **Table 2**) or antagonists (compounds 3 and 6 from **Table 2**). Among the non-similar ligands, the cyclic structure α-hexyl cinnamaldehyde evoked large responses indicating that it is an effective agonist of this receptor. We separately docked this ligand into the MOR42-3 receptor model and present its best conformation within the ligand binding pocket in **Figure 6A**. We observed hydrogen bonding between the carbonyl oxygen from α-hexyl cinnamaldehyde and the guanidinium group of arginine R179 in extracellular loop 2 (**Figure 6A**). The receptor residues I112, V113 and C116 from TMIII, L198, N199, V202, G203 and L204 from TMV and I264 from TMVI are labeled, as they represent neighboring residues within the 5Å from the docked α-hexyl cinnamaldehyde together with R179 (**Figure 6B**). The novelty of the α-hexyl cinnamaldehyde structure prompted us to look for additional ligands among structurally similar compounds. We selected and tested 4 structurally similar compounds at 100 μM. All compounds were able to activate the receptor, with similar (α-amyl cinnamaldehyde) or lower (2-butyl-1-indanone, α-methyl-trans-cinnamaldehyde, trans-cinnamaldehyde) activity than α-hexyl cinnamaldehyde (**Figure 6C**).

## Discussion

Here, we performed *in vitro* validation of *in silico* results by *in vitro* testing of 40 odorant compounds. We experimentally confirmed 22 ligands for MOR42-3, 19 agonists and 3 antagonists (underlined in **Figures 1B** and **2A**). The high positive predictive value obtained in this study results from the use of a high quality receptor model and the high accuracy in binding prediction of the ICM-VLS docking software. Six of the 19 identified agonists were known from our previous work, [5]. The previously known antagonist, dodecanedioic acid, was identified, as were two new antagonists, 2-phenyl-ethyl-2-phenylenethanoate and ethyl-3-methyl-3-phenyl-oxirane-2-carboylate (compounds 3 and 6 from **Table 2**). Our results demonstrate that cyclic structures can also activate MOR42-3. It is interesting to note that α-hexyl cinnamaldehyde has been previously identified as an agonist for human vomeronasal type 1 receptor, hVN1R1 [24], raising the interesting possibility that it is a human pheromone. There are other examples of olfactory and vomeronasal receptors sharing the same ligands, e.g. 2-heptanone can activate olfactory receptor Olfr154 and pheromone receptor V1rb2 [24], [25].

The top-ranked predicted compounds that did not show either agonist or antagonist activity in the *in vitro* assay, docked higher up in the receptor cavity (data not shown) and have a much smaller molecular volume than the validated agonists with similar hydrophobicity, indicating that a combination of the “right” molecular volume and hydrophobicity is a “sine qua non” for activation of the MOR42-3 receptor. ICM software [19] has been used previously to identify novel antagonists for a variety of receptors, such as the thyroid hormone receptor [26], the EGF receptor [27], the α-retinoic acid receptor [28] and the adenosine A2A receptor [29]. This is the first time, however, that agonists for a GPCR receptor have been identified using a VLS strategy and ICM software. Automatic modeling of mammalian olfactory receptors and docking of odorants using VINA software [30] have been proposed recently, however as of now we are not aware of any such study [31]. Our virtual ligand screening approach yielded a high positive predictive value. Determining whether this is the result of a sound 3D homology model, a highly accurate prediction algorithm of the ICM software or a relatively broad receptive range for the MOR42-3 (or a combination of these factors), must await future studies on other olfactory receptors (both those considered narrowly and broadly tuned) using the same methodology and approach.

Among the many molecular properties observed in our validated compounds, hydrophobicity clearly stood out as being very important for receptor activation. Here, we show that amino acid residues within close proximity of the docked α-hexyl cinnamaldehyde are predominantly hydrophobic (I112, V113, L198, V202, G203, L204 and I 264) further strengthening the view that non-polar, hydrophobic interaction is the predominant feature in olfactory receptor-ligand binding. The observation that odorant binding is dominated by hydrophobic contacts have also been confirmed for human hOR1G1 [32] and for mOR-EG receptor [33]. Our results also show hydrogen bonding between docked α-hexyl cinnamaldehyde and arginine R179 in extracellular loop 2 (EC2), indicating potential importance of EC2 loop in the receptor activation. Recently, Baud et al. [33] demonstrated that mutation of Phe182 in EC2 abolished mOR-EG receptor activation supporting the importance of EC2 in ligand binding and receptor activation.

The ligands identified in this study show significant structural diversity, suggesting plasticity within the ligand binding pocket and thus a broader molecular receptive range for MOR42-3 that had been previously suspected. A recent comprehensive study of MOR256-17 receptor using *in vitro* approach revealed that this particular receptor is able to detect odorants scattered across a large portion of odor space, confirming that it is broadly tuned [16]. As discussed by Charlier et al. [32], the predominant interaction between olfactory receptors and their ligands is hydrophobic. It favors multiple binding modes or conformations of the ligands within the binding pocket, which ultimately results in their broadly tuning.

Olfactory receptors, like all GPCRs, exist in equilibrium of inactive and active states, which are likely reflected in conformational changes and rearrangements of helixes III, V, VI and VII during ligand binding and receptor activation. For example, the conserved NPxxY motif of the intracellular portion of helix VII undergoes marked backbone rearrangement during GPCR receptor activation [34] and is present in MOR42-3 (NPIIY) suggesting that similar activation process occurs within olfactory receptors. On the other hand, the rearrangement of helixes VI and V by a toggle switch mechanism which involves tilting and rotation of the intracellular helix VI [35] which has been documented for A_2A_AR, opsin and β_2_AR [34], [36], [37] probably does not occur in the olfactory receptor MOR42-3 since it lacks the conserved Pro in the transmembrane helix VI necessary for induction of “toggle switch” (Pro 267^P6.50^ in Rho, and Pro 288^P6.50^ in β_2_AR). Future studies are necessary to unravel conformational changes during receptor activation and reshaping of ligand binding pocket in olfactory receptors. It would be interesting to see whether movements of the helixes of a particular olfactory receptor are dependent of ligand type.

## Supporting Information

Figure S1 The sequence alignment of rhodopsin (1U19) and mouse olfactory receptor (MOR42-3) is presented. Transmembrane domains are underlined (TM1 Q25-S55; TM2 M63-S90; TM3 L102-C131; TM4 G142-V165; TM5 L191-L216; TM6 A237-A272; TM7 L279-R299).
(TIF)

Figure S2 PDB file of MOR42-3 receptor.
(PDB)

Figure S3 A histogram showing a normal distribution of frequency of binned docking scores.
(TIF)

Table S1 574 odorants ranked by the VLS score function. Explicit hydrogens are not shown in the molecular structure. mfScore for each compound is shown in the last column. Abbreviations used for molecular descriptors: molLogP is the log of the octanol/water ratio; molPSA is the molecular polar surface area; Score is the binding energy score; Natom is the number of atoms in docked ligand; Nflex is the number of rotatable torsions; Hbond is Hydrogen Bond energy; Hphob is the hydrophobic energy in exposing a surface to water; VwInt is the van der Waals interaction energy; Eintl is internal conformation energy of the ligand; Dsolv is the desolvation of exposed h-bond donors and acceptors; SolEl is the solvation electrostatics energy change upon binding and mfScore is the potential of mean force score.
(PDF)
