# Supplementary figures and images for: Discovery of Novel Ligands for Mouse Olfactory Receptor MOR42-3 Using an In Silico Screening Approach and In Vitro Validation
Source: PLoS One. 2014 Mar 17;9(3):e92064. doi: 10.1371/journal.pone.0092064 (PMC3956865; doi:10.1371/journal.pone.0092064)

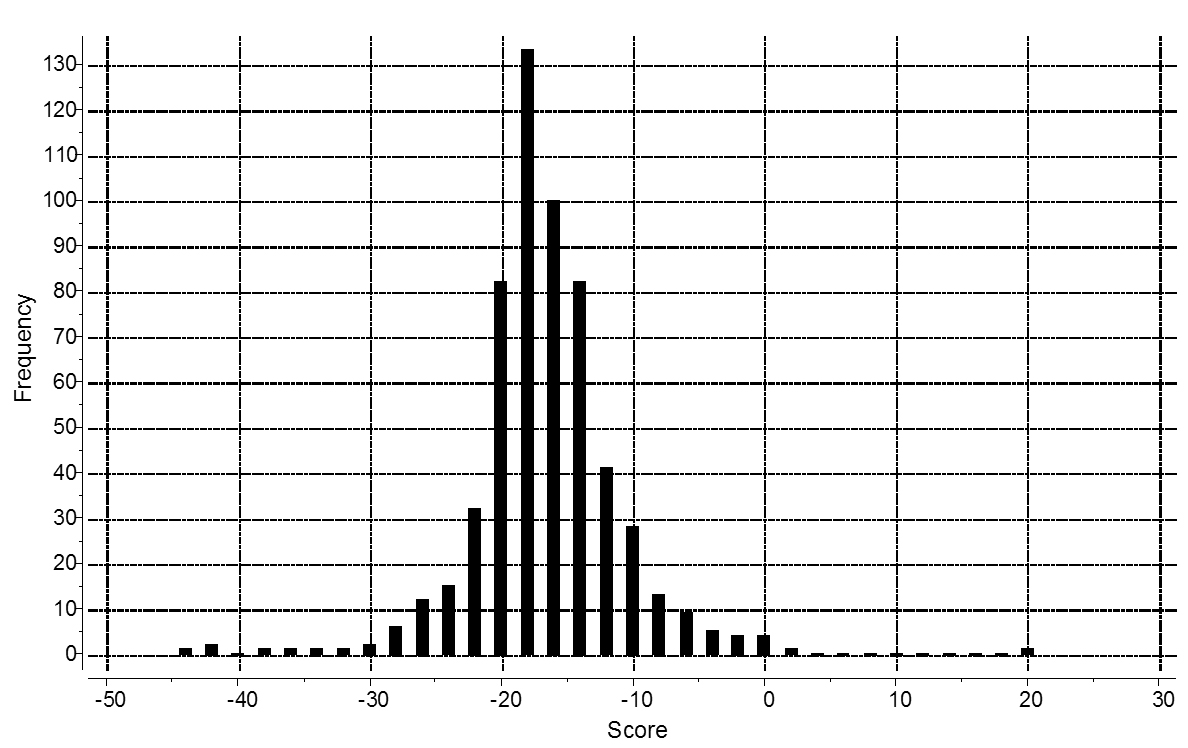

Supplement: Figure S3 — A histogram showing a normal distribution of frequency of binned docking scores. (TIF) [file pone.0092064.s003.tif]
