# Supplementary material for: Discovery of Novel Ligands for Mouse Olfactory Receptor MOR42-3 Using an In Silico Screening Approach and In Vitro Validation
Source: PLoS One. 2014 Mar 17;9(3):e92064. doi: 10.1371/journal.pone.0092064 (PMC3956865; doi:10.1371/journal.pone.0092064)
Supplement: Table S1 — 574 odorants ranked by the VLS score function. Explicit hydrogens are not shown in the molecular structure. mfScore for each compound is shown in the last column. Abbreviations used for molecular descriptors: molLogP is the log of the octanol/water ratio; molPSA is the molecular polar surface area; Score is the binding energy score; Natom is the number of atoms in docked ligand; Nflex is the number of rotatable torsions; Hbond is Hydrogen Bond energy; Hphob is the hydrophobic energy in exposing a surface to water; VwInt is the van der Waals interaction energy; Eintl is internal conformation energy of the ligand; Dsolv is the desolvation of exposed h-bond donors and acceptors; SolEl is the solvation electrostatics energy change upon binding and mfScore is the potential of mean force score. (PDF) [file pone.0092064.s004.pdf]

Rank	mol	Name	formula	molWeight	Score	molLogP	molPSA	molVolume	Natom	Nflex	Hbond	Hphob	VwInt	Eintl	Dsolv	SolEl	mfScore
1	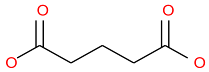	glutaric acid	C5H8O4	132.042	-42.01	-0.05	57.28	121.52	15	4	-13.27	-1.49	-4.45	0.81	8.75	-18.1	-11.49
2	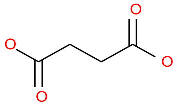	succinic acid	C4H6O4	118.027	-40.81	-0.53	57.28	103.62	12	3	-13.21	-1.21	-8.03	0	8.75	-10.45	-25.76
3	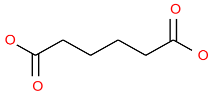	adipic acid	C6H10O4	146.058	-40.15	0.43	57.28	139.43	18	5	-12.18	-1.87	-8.76	5.58	8.68	-13.4	-20.05
4	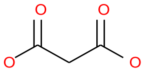	malonic acid	C3H4O4	104.011	-36.55	-0.96	57.28	85.29	9	2	-9.82	-0.74	-12.03	0.65	8.67	-10.43	-18.27
5	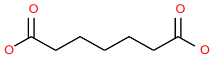	heptanedioic acid	C7H12O4	160.074	-35.61	0.92	57.28	157.34	21	6	-12.42	-2.19	-2.57	7.58	8.73	-15.59	-4.98
6	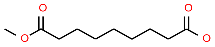	9-methoxy-9-oxo-nonanoic acid	C10H18O4	202.121	-32.99	2.23	49.47	216.15	31	8	-10.57	-4.8	-14.41	0.51	12.26	-3.95	-76.98
7	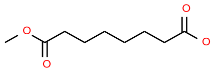	8-methoxy-8-oxo-octanoic acid	C9H16O4	188.105	-30.2	1.75	49.47	198.25	28	7	-9.31	-4.27	-17.49	2.66	12.59	0.08	-98.29
8	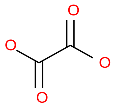	oxalic acid	C2H2O4	89.995	-29.72	-0.54	57.33	67.91	6	1	-10.84	-0.3	-7.21	0.01	8.63	-2.99	-30.76
9	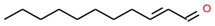	undec-2-enal	C11H20O	168.151	-28.66	4.35	14.61	215.74	32	7	-7.02	-5.31	-21.55	8.39	8.05	6.01	-103.73
10	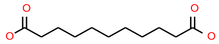	undecanedioic acid	C11H20O4	216.136	-27.67	2.84	57.28	228.96	33	10	-10.3	-4.45	-15.11	4.96	13.15	0.95	-104.3
11	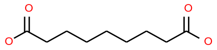	nonanedioic acid	C9H16O4	188.105	-27.5	1.88	57.28	193.15	27	8	-9.26	-3.6	-15.77	4.6	11.98	0.51	-97.71
12	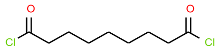	nonanedioyl chloride	C9H14Cl2O2	224.037	-26.89	3.41	27.2	215.39	27	8	-7.95	-5.2	-15.22	0.97	8.86	0.73	-84.05
13	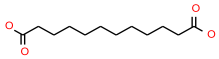	dodecanedioic acid	C12H22O4	230.152	-26.78	3.33	57.28	246.87	36	11	-10.29	-4.75	-14.3	8.79	13.02	0.55	-105.11
14	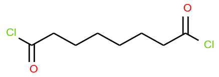	octanedioyl chloride	C8H12Cl2O2	210.021	-26.75	2.93	27.2	197.48	24	7	-6.86	-4.86	-20.67	0	8.89	5.54	-88.72

15	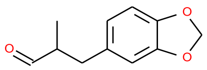	3-(7,9-dioxa-bicyclo[4.3.0]nona-1,3,5-trien-3-yl)-2-methyl-propanal	C11H12O3	192.079	-26	2.44	30.69	198.9	26	3	-5.39	-4.55	-20.01	0.86	10.5	1.56	-89.87
16	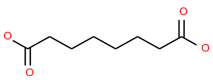	octanedioic acid	C8H14O4	174.089	-25.29	1.4	57.28	175.24	24	7	-11.44	-2.58	3.33	4.63	8.46	-12.88	-10.78
17	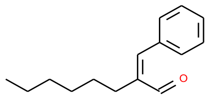	alpha-hexyl cinnamaldehyde	C15H22O	216.151	-25.24	4.98	14.4	255.25	36	6	-7.27	-5.93	-16.17	1.27	8.88	4.58	-53.42
18	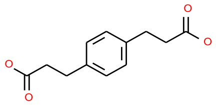	3-(4-(2-carboxy-ethyl)-phenyl)-propanoic acid	C12H14O4	222.089	-24.98	1.81	57.01	214.27	28	6	-9.94	-3.99	-14.31	1.73	12.95	5.83	-102.9
19	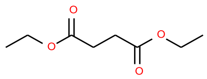	1,4-diethyl butanedioate	C8H14O4	174.089	-24.57	1.12	40.83	183.64	26	5	-7.23	-4.17	-11.23	3.13	7.92	-1.83	-41.03
20	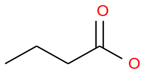	butyric acid	C4H8O2	88.052	-24.49	0.87	28.89	91.05	13	2	-6.83	-2.04	-6.89	0.68	5.02	-5.93	-5.17
21	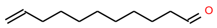	undec-10-enal	C11H20O	168.151	-24.27	3.96	14.61	216.02	32	9	-5	-5.39	-22.33	3.14	7.65	4.81	-88.66
22	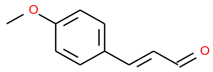	3-(4-methoxy-phenyl)-acrylaldehyde	C10H10O2	162.068	-24.26	2.38	21.89	175.81	22	0	-4.93	-4.14	-17.57	0.81	8.48	3.33	-84.61
23	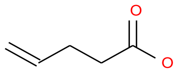	pent-4-enoic acid	C5H8O2	100.052	-24.2	0.97	28.89	112.8	14	3	-6.32	-2.4	-10.29	2.04	5.2	-3.03	-8.3
24	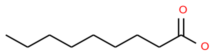	nonanoic acid	C9H18O2	158.131	-24.19	3.28	28.89	180.58	28	7	-6.56	-4.62	-16.34	3.58	9.51	0.77	-110.39
25	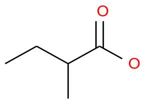	2-methyl-butanoic acid	C5H10O2	102.068	-24.18	1.19	28.92	106.24	16	2	-6.96	-2.53	-5.95	1.13	5.19	-6.1	-11.79
26	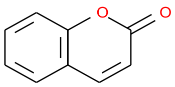	3-oxo-2-oxa-bicyclo[4.4.0]deca-1(6),4,7,9-tetraene	C9H6O2	146.037	-24.04	1.72	20.65	151.42	17	0	-6.55	-3.41	-13.62	0	7.94	3.75	-64.12
27	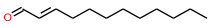	dodec-2-enal	C12H22O	182.167	-23.7	4.83	14.61	233.64	35	8	-4.82	-5.71	-21.69	10.21	8.05	4.81	-105.73
28	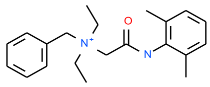	2-(diethyl-(phenyl-methyl)-amino)-1-(2,6-dimethyl-phenylamino)-ethanone	C21H29N2O	446.257	-23.57	4.91	50.12	461.02	53	6	-2.58	-6.54	-18.89	1.59	1.29	2.49	-71.55
29	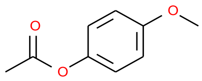	4-methoxy-phenyl ethanoate	C9H10O3	166.063	-22.85	1.68	28.53	167.45	22	0	-4.2	-4.36	-18.67	0	9.71	3.16	-97.52

30	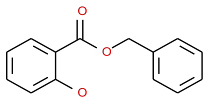	phenyl-methyl 2-hydroxy-benzoate	C14H12O3	228.079	-22.84	3.15	37.64	215.23	29	2	-3.09	-4.83	-15.95	0	4.08	-0.15	-56.9
31	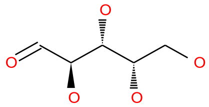	(2R,3R,4S)-2,3,4,5-tetrahydroxy-pentanal	C5H10O5	150.053	-22.84	-3	79.77	126.4	20	8	-15.85	-1.21	-6.83	3.44	15.61	12.7	-0.96
32	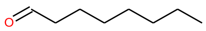	octanal	C8H16O	128.12	-22.76	2.9	14.61	158.45	25	6	-4.96	-4.17	-17.6	1.71	6.92	2.1	-89.01
33	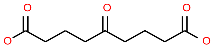	5-oxo-nonanedioic acid	C9H14O5	202.084	-22.74	0.46	70.03	202.57	26	8	-14.24	-3.12	-0.16	0.38	13.34	1.1	-68.79
34	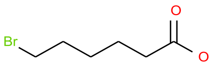	6-bromo-hexanoic acid	C6H11BrO2	193.994	-22.67	2.16	28.89	146	19	5	-5.67	-3.88	-15.22	1.1	8.89	-0.26	-85.73
35	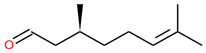	(S)-3,7-dimethyl-oct-6-enal	C10H18O	154.136	-22.49	3.37	14.61	198.02	29	5	-5.02	-4.9	-19.01	0	7.97	5.37	-91.96
36	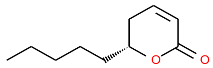	(R)-6-oxo-2-pentyl-3,6-dihydro-2H-pyran	C10H16O2	168.115	-22.46	2.78	21.06	205.58	28	4	-4.44	-4.97	-19.67	1.65	8.97	4	-94.46
37	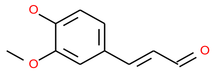	3-(4-hydroxy-3-methoxy-phenyl)-acrylaldehyde	C10H10O3	178.063	-22.46	2	38.52	187.24	23	0	-4.06	-3.95	-21.01	0.79	9.74	6.31	-92.1
38	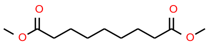	1,9-dimethyl nonanedioate	C11H20O4	216.136	-22.29	2.58	41.67	239.16	35	8	-7.05	-5.69	-16.2	3.49	12.09	2.57	-82.82
39	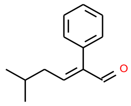	5-methyl-2-phenyl-hex-2-enal	C13H16O	188.12	-22.18	3.71	14.4	216.98	30	3	-2.61	-4.98	-23.58	1.27	8.07	5.31	-121.47
40	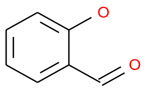	2-hydroxy-benzaldehyde	C7H6O2	122.037	-22.08	1.27	30.95	114.6	15	0	-3.77	-2.46	-15.73	2.78	5.41	1.18	-77.2
41	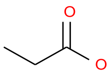	propionic acid	C3H6O2	74.037	-22.03	0.39	28.89	73.58	10	1	-6.51	-1.68	-9.81	0	4.99	1.55	-12.46
42	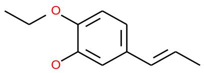	2-ethoxy-5-(prop-1-enyl)-phenol	C11H14O2	178.099	-21.67	3.39	24	197.13	27	1	-6.59	-5.22	-11.56	0	9.26	3.81	-77.3
43	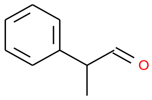	2-phenyl-propanal	C9H10O	134.073	-21.63	1.73	14.4	134.9	20	2	-4.85	-3.68	-18.38	0.05	7.1	7.52	-97.73
44	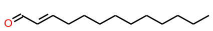	tridec-2-enal	C13H24O	196.183	-21.62	5.31	14.61	251.55	38	9	-6.99	-6.12	-15.25	6.62	8.08	5.99	-104.79

45	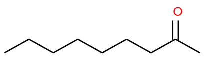	nonan-2-one	C9H18O	142.136	-21.58	3.25	13.84	176.66	28	6	-5.07	-5	-15.72	1.19	6.82	2.42	-81.7
46	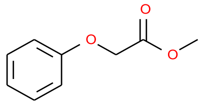	methyl 2-phenoxy-ethanoate	C9H10O3	166.063	-21.49	1.55	28.32	161.34	22	3	-5.83	-4.3	-11.89	2.57	7.66	0.72	-44.59
47	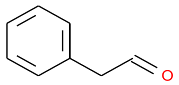	2-phenyl-acetaldehyde	C8H8O	120.058	-21.44	1.51	14.34	124.59	17	2	-4.93	-3.24	-15.5	0.07	6.54	3.92	-66.84
48	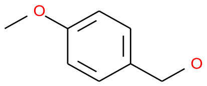	(4-methoxy-phenyl)-methanol	C8H10O2	138.068	-21.18	1.37	24.9	135.9	20	2	-2.65	-3.52	-14.65	1.25	4.27	-2.83	-30.83
49	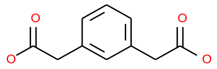	2-(3-(carboxy-methyl)-phenyl)-acetic acid	C10H10O4	194.058	-21.12	0.95	57.01	178.84	22	4	-4.85	-1.82	-10.59	1.02	7.55	-7.74	-50.83
50	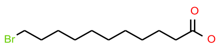	11-bromo-undecanoic acid	C11H21BrO2	264.072	-21.09	4.57	28.89	235.53	34	10	-6.91	-5.81	-13.27	9.14	9.7	0.42	-96.19
51	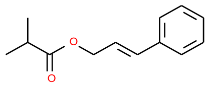	3-phenyl-allyl 2-methyl-propanoate	C13H16O2	204.115	-21	3.42	21.29	224.76	31	4	-3.59	-5.77	-16.45	2.3	5.18	3.22	-29.17
52	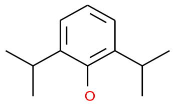	2,6-diisopropyl-phenol	C12H18O	178.136	-20.98	3.59	15.72	195.09	31	2	-2.49	-5.14	-13.1	1.13	1.98	-1.25	-39
53	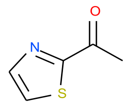	1-(thiazol-2-yl)-ethanone	C5H5NOS	127.009	-20.91	0.76	24.5	109.15	13	0	-7.14	-2.78	-13.62	0.15	4.86	13.64	-4.04
54	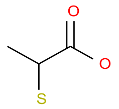	2-mercapto-propanoic acid	C3H6O2S	106.009	-20.9	0.37	28.92	89.44	11	2	-7.22	-1.3	-9.84	1.19	6.78	2.71	-27.77
55	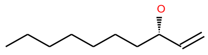	(S)-dec-1-en-3-ol	C10H20O	156.151	-20.84	3.77	17.13	193.6	31	8	-3.78	-5.46	-22.15	5.46	9.33	4.13	-97.94
56	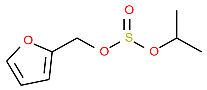	furan-2-ylmethyl isopropyl sulfite	C8H12O4S	204.243	-20.8	0.96	39.31	169.25	25	5	-5.86	-4.79	-17.58	1.24	11.54	4.79	-93.2
57	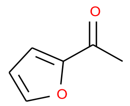	1-(furan-2-yl)-ethanone	C6H6O2	110.037	-20.75	0.83	22.64	107.73	14	0	-7.02	-2.71	-13.99	0.22	4.73	14.04	-3.57
58	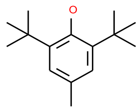	4-methyl-2,6-ditert-butyl-phenol	C15H24O	220.183	-20.64	4.99	15.72	277.97	40	2	0	-6.27	-16.37	0	-0.24	-1.66	-70.29
59	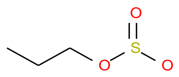	propyl hydrogen sulfite	C3H8O3S	124.159	-20.6	-0.24	39.46	95.44	14	3	-7.05	-2.11	-12.39	0.2	6.64	6.46	-6.39

60	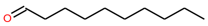	decanal	C10H20O	156.151	-20.55	3.87	14.61	194.27	31	8	-4.92	-4.98	-17.85	3.36	7.69	3.83	-89.79
61	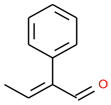	2-phenyl-but-2-enal	C10H10O	146.073	-20.51	2.51	14.4	166.87	21	1	-2.67	-4	-19.14	0	7.48	2.82	-83.04
62	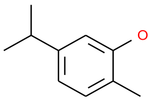	5-isopropyl-2-methyl-phenol	C10H14O	150.104	-20.48	3	16.79	160.73	25	1	-2.4	-4.6	-11.89	3.17	2.51	-2.92	-37.03
63	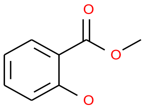	methyl 2-hydroxy-benzoate	C8H8O3	152.047	-20.43	1.53	37.96	143.66	19	0	-4.27	-3.5	-14.65	1.53	7.38	2.79	-74.03
64	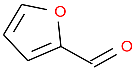	furan-2-carbaldehyde	C5H4O2	96.021	-20.42	0.87	22.15	90.05	11	0	-6.67	-1.84	-16.48	0.05	5	15.78	-19.42
65	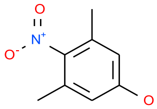	3,5-dimethyl-4-nitrophenol	C8H9NO3	167.162	-20.37	1.83	50.64	153.25	21	1	-2.76	-3.51	-13.73	0	3.16	-0.43	-44.45
66	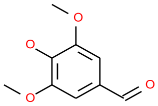	4-hydroxy-3,5-dimethoxy-benzaldehyde	C9H10O4	182.058	-20.35	1.33	45.14	178.02	23	0	-6.48	-3.42	-15.92	0	10.74	9.39	-53.71
67	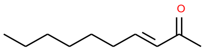	dec-3-en-2-one	C10H18O	154.136	-20.25	3.47	13.84	201.86	29	5	-2.89	-5.23	-20.24	0	7.38	3.43	-82.71
68	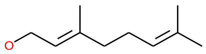	(2E)-3,7-dimethyl-octa-2,6-dien-1-ol	C10H18O	154.136	-20.25	3.33	17.17	210.16	29	5	-2.77	-4.67	-15.46	0	3.19	-0.08	-30.79
69	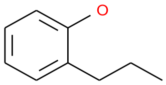	2-propyl-phenol	C9H12O	136.089	-20.22	2.86	16.79	144.04	22	2	-2.85	-4.09	-12.89	0.75	2.48	-0.66	-35.78
70	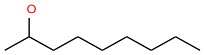	nonan-2-ol	C9H20O	144.151	-20.2	3.51	16.99	173.48	30	7	-4.22	-5.03	-18.87	5.2	8.91	2.78	-107.95
71	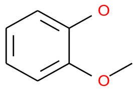	2-methoxy-phenol	C7H8O2	124.052	-20.1	1.57	24.42	119.09	17	0	-2.36	-3.5	-11.75	0.46	1.1	-1.29	-19.76
72	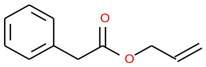	allyl 2-phenyl-ethanoate	C11H12O2	176.084	-20.08	2.5	20.69	189.42	25	4	-2.05	-5.13	-24.7	1.97	8.65	6.62	-114.24
73	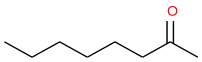	octan-2-one	C8H16O	128.12	-20.06	2.77	13.84	158.75	25	5	-5.3	-4.73	-13.01	0	6.87	2.11	-72.6
74	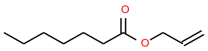	allyl heptanoate	C10H18O2	170.131	-19.94	3.41	20.96	206.86	30	7	-3.93	-5.59	-20.15	1.06	9.07	4.38	-115.77

75	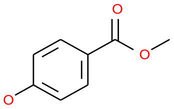	methyl 4-hydroxy-benzoate	C8H8O3	152.047	-19.91	1.65	39.03	143.03	19	0	-5.22	-3.48	-14.83	0.37	10.39	4.06	-66.85
76	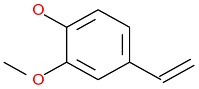	2-methoxy-4-vinyl-phenol	C9H10O2	150.068	-19.85	2.43	24.42	163.95	21	0	-4.67	-4.32	-17.37	0	10.11	7.03	-83.82
77	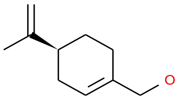	(S)-(4-isopropenyl-cyclohex-1-enyl)-methanol	C10H16O	152.12	-19.81	2.85	17.55	198.5	27	3	-3.03	-3.96	-13.51	1.2	3.25	-0.33	-26.72
78	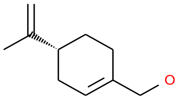	(R)-(4-isopropenyl-cyclohex-1-enyl)-methanol	C10H16O	152.12	-19.81	2.85	17.55	198.5	27	3	-3.03	-3.95	-13.48	1.21	3.25	-0.4	-26.58
79	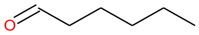	hexanal	C6H12O	100.089	-19.79	1.94	14.61	122.64	19	4	-4.66	-3.29	-14.05	0.41	6.11	1.91	-80.11
80	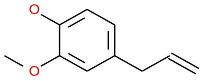	4-allyl-2-methoxy-phenol	C10H12O2	164.084	-19.77	2.61	24.42	181.27	24	2	-1.02	-4.64	-17.56	0.46	3.93	-1.01	-45.95
81	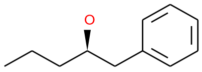	(R)-1-phenyl-pentan-2-ol	C11H16O	164.12	-19.77	3.12	16.86	173	28	5	-2.65	-4.75	-14.18	1.14	2.92	-1.36	-38.52
82	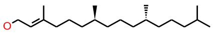	(7S,11R,E)-3,7,11,15-tetramethyl-hexadec-2-en-1-ol	C20H40O	296.308	-19.74	8.28	17.17	372.58	61	14	-2.51	-6.05	-22.19	7.04	6.02	-0.09	-75.56
83	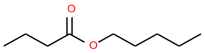	pentyl butanoate	C9H18O2	158.131	-19.74	3.14	20.96	185.28	29	6	-3.78	-5.06	-13.73	3.86	5.97	-1.73	-53.8
84	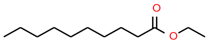	ethyl decanoate	C12H24O2	200.178	-19.7	4.59	20.67	238.5	38	9	-4.05	-6.49	-20.52	5.31	9.25	4.66	-85.31
85	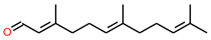	3,7,11-trimethyl-dodeca-2,6,10-trienal	C15H24O	220.183	-19.63	5.48	14.61	309.85	40	6	0	-4.79	-21.92	0	2.65	-0.14	-60.27
86	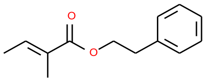	2-phenyl-ethyl 2-methyl-but-2-enoate	C13H16O2	204.115	-19.56	3.51	21.29	230.67	31	3	0	-5.53	-17.44	0	0.66	-1.08	-44.87
87	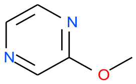	2-methoxy-pyrazine	C5H6N2O	110.048	-19.55	0.11	26.25	97.1	14	0	-7.37	-2.73	-15.51	0.06	6.09	17.99	-12.82
88	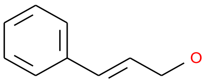	3-phenyl-prop-2-en-1-ol	C9H10O	134.073	-19.53	2.12	16.89	145.12	20	3	-4.14	-4.23	-14.8	0.2	8.72	0.39	-75.8
89	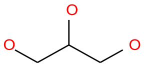	propane-1,2,3-triol	C3H8O3	92.047	-19.53	-1.61	51.36	81.67	14	5	-11.88	-1.54	-10.61	0.68	11.15	16.35	-10.42

90	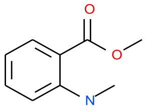	methyl 2-methylamino-benzoate	C9H11NO2	165.079	-19.5	1.63	31.37	158.05	23	0	-0.76	-4.09	-15.44	0	1.11	-0.37	-43.85
91	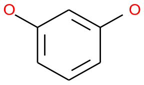	resorcinol	C6H6O2	110.037	-19.43	1.34	35.47	97.07	14	0	-4.42	-2.28	-11.63	1.12	5.82	0.85	-32.3
92	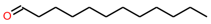	dodecanal	C12H24O	184.183	-19.39	4.83	14.61	230.08	37	10	-3.55	-5.83	-22.44	8.5	8.05	5.92	-120.81
93	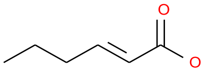	hex-2-enoic acid	C6H10O2	114.068	-19.39	1.98	28.89	133.84	17	3	-5.49	-2.73	-11.85	3.28	5.25	3.71	-3.02
94	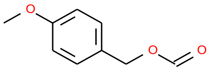	((4-methoxy-phenyl)-methoxy)-methanone	C9H10O3	166.063	-19.3	1.63	30.11	161.45	22	2	-3.23	-3.87	-19.7	0.23	9.85	3.91	-86.62
95	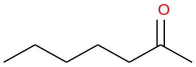	heptan-2-one	C7H14O	114.104	-19.28	2.29	13.84	140.84	22	4	-5	-4.3	-13.42	0	6.82	3.26	-64.9
96	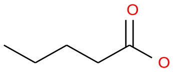	pentanoic acid	C5H10O2	102.068	-19.25	1.35	28.89	108.96	16	3	-5.65	-2.44	-10.56	1.46	5.22	2.33	-5.22
97	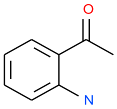	1-(2-amino-phenyl)-ethanone	C8H9NO	135.068	-19.21	1.04	34.16	128.19	19	0	-4.05	-3.26	-12.98	0	6.26	2.28	-84.16
98	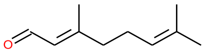	3,7-dimethyl-octa-2,6-dienal	C10H16O	152.12	-19.21	3.49	14.61	210.36	27	3	0	-4.07	-19.59	0	1.79	-0.07	-39.78
99	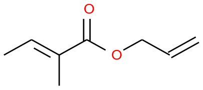	allyl 2-methyl-but-2-enoate	C8H12O2	140.084	-19.2	2.34	21.56	180.31	22	2	0	-4.63	-16.42	0	1.19	-2.69	-23.87
100	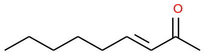	non-3-en-2-one	C9H16O	140.12	-19.18	2.99	13.84	183.95	26	4	-3.77	-4.84	-14.34	0	4.68	3.08	-62
101	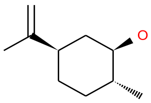	(1R,2R,5R)-5-isopropenyl-2-methyl-cyclohexanol	C10H18O	154.136	-19.14	2.79	16.05	185.65	29	2	-2.75	-4.6	-11.23	0	2.44	-1.36	-29.37
102	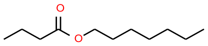	heptyl butanoate	C11H22O2	186.162	-19.12	4.11	20.96	221.09	35	8	-3.86	-6.2	-16.73	4.92	8.47	0.57	-69.64
103	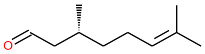	(R)-3,7-dimethyl-oct-6-enal	C10H18O	154.136	-19.1	3.37	14.61	198.02	29	5	-2.93	-4.61	-20.04	0	7.63	4.11	-90.23
104	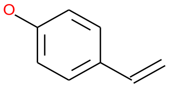	4-vinyl-phenol	C8H8O	120.058	-19.07	2.46	17.86	131.23	17	0	-2.9	-3.23	-12.24	0.55	3.17	0.46	-37.96

105	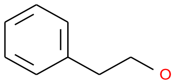	2-phenyl-ethanol	C8H10O	122.073	-19.03	1.6	16.89	122.59	19	3	-2.86	-3.45	-13.63	0.01	2.91	0.26	-41.49
106	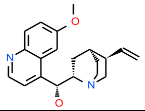	(1R)-(6-methoxyquinolin-4-yl)((2S,4S,5R)-5-vinylquinuclidin-2-yl)methanol	C20H24N2O2	324.184	-19.02	2.41	35.65	339.59	48	4	-2.43	-5.9	-14.63	1.31	3.72	0.79	-52.76
107	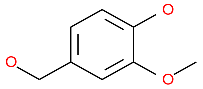	4-(hydroxy-methyl)-2-methoxy-phenol	C8H10O3	154.063	-19.02	0.99	41.53	147.33	21	2	-3.1	-3.65	-13.52	1.45	3.55	1.4	-49.39
108	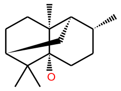	(1R,4R,4aR,6R,8aS)-4,8a,9,9-tetramethyldecahydro-1,6-methanonaphthalen-1-ol	C15H26O	222.366	-19.02	4.33	15.18	288.51	42	1	-2.85	-4.83	-9.72	0	2.58	-2.09	-41.6
109	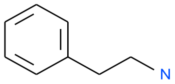	2-phenyl-ethanamine	C8H11N	121.089	-19.02	1	21.39	124.65	20	3	-2.94	-3.42	-14.57	0.03	3.84	0.89	-39.31
110	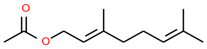	3,7-dimethyl-octa-2,6-dienyl ethanoate	C12H20O2	196.146	-19.01	3.9	21.66	257.82	34	5	-3.67	-5.8	-19.06	0	9.57	4.61	-71.09
111	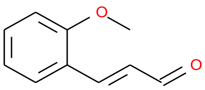	3-(2-methoxy-phenyl)-acrylaldehyde	C10H10O2	162.068	-18.98	2.26	21.97	174.49	22	0	0	-4.22	-16.44	0.42	1.76	-1.55	-39.63
112	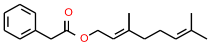	3,7-dimethyl-octa-2,6-dienyl 2-phenyl-ethanoate	C18H24O2	272.178	-18.96	5.41	20.69	328.95	44	7	0	-6.09	-22.07	0	1.24	3.11	-98.94
113	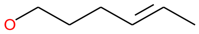	hex-4-en-1-ol	C6H12O	100.089	-18.96	1.69	17.17	125.51	19	4	-4.64	-3.65	-14.65	0	7.69	2.57	-67.5
114	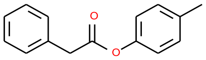	4-methyl-phenyl 2-phenyl-ethanoate	C15H14O2	226.099	-18.96	3.51	20.02	227.67	31	2	-2.86	-5.8	-22.17	0	9.63	8.96	-65.82
115	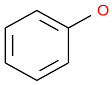	phenol	C6H6O	94.042	-18.96	1.6	17.86	86.44	13	0	-4.36	-2.75	-11.79	0.1	5.16	2.77	-56.38
116	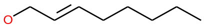	oct-2-en-1-ol	C8H16O	128.12	-18.84	2.74	17.17	163.17	25	6	-3.62	-4.56	-18.59	0.55	8.5	3.04	-86.54
117	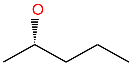	(S)-pentan-2-ol	C5H12O	88.089	-18.79	1.58	16.99	101.85	18	3	-4.25	-3.35	-10.44	0.35	5.28	-1.71	-63.84
118	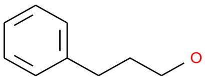	3-phenyl-propan-1-ol	C9H12O	136.089	-18.79	2.08	16.89	140.5	22	4	-2.68	-3.71	-13.61	0.59	2.82	-0.67	-43.49
119	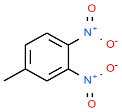	4-methyl-1,2-dinitro-benzene	C7H6N2O4	182.033	-18.77	1.59	76.16	149.24	19	2	0	-3.24	-14.43	0	0	-5.1	-36.24

120	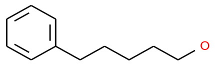	5-phenyl-pentan-1-ol	C11H16O	164.12	-18.74	3.04	16.89	176.31	28	6	-3.89	-5.06	-19.31	2.27	9.17	5.02	-96.16
121	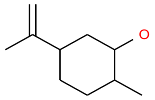	5-isopropenyl-2-methyl-cyclohexanol	C10H18O	154.136	-18.73	2.79	16.05	185.65	29	2	-2.82	-4.2	-10.88	0	2.84	-1.85	-32.24
122	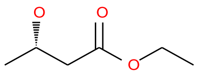	(S)-ethyl 3-hydroxy-butanoate	C6H12O3	132.079	-18.72	0.53	37.15	135.59	21	4	-5.52	-3.53	-13.2	1.63	8.99	2.54	-60.74
123	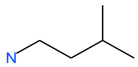	3-methyl-butan-1-amine	C5H13N	87.105	-18.62	0.83	21.67	103.75	19	3	-4.3	-3.1	-13.04	2.52	7.12	0.37	-61.39
124	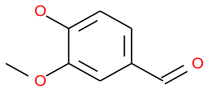	4-hydroxy-3-methoxy-benzaldehyde	C8H8O3	152.047	-18.59	1.36	38.58	147.35	19	0	-5.78	-3.18	-14.32	6.52	11.24	6.16	-72.05
125	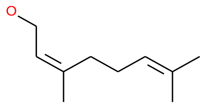	(2Z)-3,7-dimethyl-octa-2,6-dien-1-ol	C10H18O	154.136	-18.56	3.33	17.17	210.16	29	5	-4.1	-4.98	-18.25	0	9.27	5.22	-82.02
126	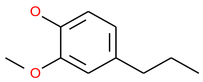	2-methoxy-4-propyl-phenol	C10H14O2	166.099	-18.52	2.95	24.42	175.79	26	2	-0.99	-4.74	-16.32	0.57	3.88	-0.96	-50.85
127	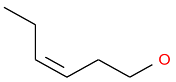	(Z)-hex-3-en-1-ol	C6H12O	100.089	-18.5	1.61	17.17	128.54	19	4	-4.11	-3.7	-14.78	0	7.85	1.3	-69.86
128	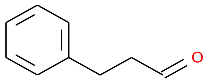	3-phenyl-propanal	C9H10O	134.073	-18.49	1.94	14.34	140.86	20	3	-4.36	-3.8	-16.65	0	7.99	6.01	-86.43
129	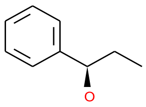	(R)-1-phenyl-propan-1-ol	C9H12O	136.089	-18.46	2.23	16.39	136.46	22	3	-4.47	-4.21	-17.17	0.09	8.91	6.6	-92.95
130	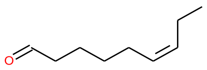	(Z)-non-6-enal	C9H16O	140.12	-18.45	2.92	14.61	182.63	26	6	-3.62	-4.3	-17.98	0.63	7.72	3.29	-90.93
131	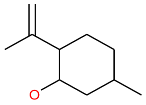	2-isopropenyl-5-methyl-cyclohexanol	C10H18O	154.136	-18.43	2.79	16.05	184.17	29	2	-2.51	-4.57	-11.85	0.45	2.64	-0.53	-33.76
132	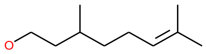	3,7-dimethyl-oct-6-en-1-ol	C10H20O	156.151	-18.42	3.51	17.17	199.03	31	6	-2.32	-4.71	-13.75	0	2.87	-2.11	-39.31
133	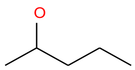	pentan-2-ol	C5H12O	88.089	-18.39	1.58	16.99	101.85	18	3	-4.26	-3.3	-11.21	0.48	5.64	-0.34	-65.26
134	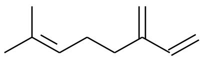	7-methyl-3-methylene-octa-1,6-diene	C10H16	136.125	-18.39	3.91	0	203.76	26	3	0	-4.75	-15.94	0	0	-1.63	-32.55

135	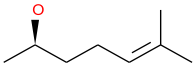	(R)-6-methyl-hept-5-en-2-ol	C8H16O	128.12	-18.38	2.6	16.99	163.64	25	4	-4.05	-4.7	-15.17	0	8.6	2.02	-89.57
136	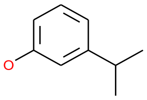	3-isopropyl-phenol	C9H12O	136.089	-18.33	2.72	17.86	139.58	22	1	-3.97	-4.22	-16.21	0.76	9	5.15	-94.21
137	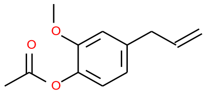	4-allyl-2-methoxy-phenyl ethanoate	C12H14O3	206.094	-18.3	2.61	28.71	229.13	29	2	0	-5.51	-16.51	0	2.05	-1.27	-41.52
138	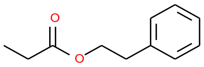	2-phenyl-ethyl propanoate	C11H14O2	178.099	-18.29	2.66	20.69	186.03	27	4	-4.06	-4.82	-12.8	3.84	6.96	0.59	-77.26
139	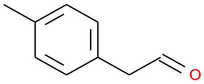	2-p-tolyl-acetaldehyde	C9H10O	134.073	-18.28	1.91	14.34	145.53	20	2	-3.51	-3.57	-14.14	0	5.26	3.02	-66.78
140	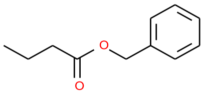	phenyl-methyl butanoate	C11H14O2	178.099	-18.28	2.84	20.77	185.62	27	4	-3.32	-5.06	-13.5	1.56	6.43	-0.34	-61.03
141	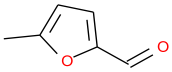	5-methyl-furan-2-carbaldehyde	C6H6O2	110.037	-18.28	1.3	21.66	111.07	14	0	-6.43	-2.31	-16.2	0	4.86	18.31	-4.64
142	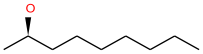	(R)-nonan-2-ol	C9H20O	144.151	-18.24	3.51	16.99	173.48	30	7	-4.19	-5.14	-16.46	3.94	9.03	1.94	-109.08
143	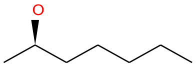	(R)-heptan-2-ol	C7H16O	116.12	-18.24	2.55	16.99	137.66	24	5	-4.24	-4.34	-15.63	1.96	8.33	2.7	-86.1
144	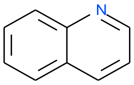	2-aza-bicyclo[4.4.0]deca-1(10),2,4,6,8-pentaene	C9H7N	129.058	-18.23	2.3	9	120.17	17	0	-3.07	-3.89	-14.97	0	6.57	3.31	-78.07
145	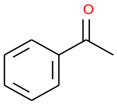	acetophenone	C8H8O	120.058	-18.23	1.61	14.07	122.3	17	1	-2.92	-3.51	-14.25	0	5.22	1.88	-65.7
146	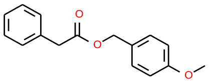	(4-methoxy-phenyl)-methyl 2-phenyl-ethanoate	C16H16O3	256.11	-18.17	3.46	28.04	253.75	35	4	-1.72	-5.6	-17.71	1.89	3.78	3.68	-46.5
147	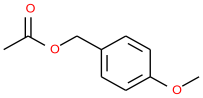	(4-methoxy-phenyl)-methyl ethanoate	C10H12O3	180.079	-18.12	1.95	29.01	182.62	25	2	-3.57	-4.74	-17.72	0	9.29	5.51	-100.75
148	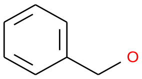	phenyl-methanol	C7H8O	108.058	-18.11	1.28	17.35	104.06	16	2	-3.68	-3.23	-13.36	0.42	6.56	0.92	-70.17
149	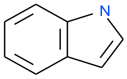	7-aza-bicyclo[4.3.0]nona-1(6),2,4,8-tetraene	C8H7N	117.058	-18.1	2.19	10.02	112.41	16	0	-2.21	-3.39	-13.68	0	3.52	1.23	-65.11

150	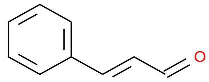	3-phenyl-acrylaldehyde	C9H8O	132.058	-18.06	2.29	14.34	143.97	18	1	-2.62	-3.75	-17.24	0	7.25	3.44	-78.17
151	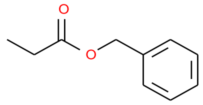	phenyl-methyl propanoate	C10H12O2	164.084	-18.04	2.35	20.77	168.16	24	3	-3.27	-4.7	-12.88	1.49	6.4	-0.51	-59.12
152	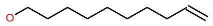	dec-9-en-1-ol	C10H20O	156.151	-18.03	3.62	17.17	197.74	31	9	-4.13	-5.4	-20.66	4.26	8.63	7.3	-83.82
153	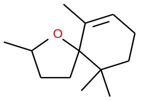	2,6,10,10-tetramethyl-1-oxa-spiro[4.5]dec-6-ene	C13H22O	194.167	-18.01	4.33	7.79	264.86	36	0	0	-5.1	-14.21	0.05	0.74	-1.44	-55.1
154	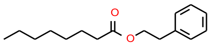	2-phenyl-ethyl octanoate	C16H24O2	248.178	-17.99	5.07	20.69	275.12	42	9	0	-5.32	-22.36	6.22	3.58	-0.24	-72.67
155	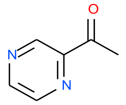	1-(pyrazin-2-yl)-ethanone	C6H6N2O	122.048	-17.95	-0.38	32.63	112.49	15	0	-8.66	-2.55	-10.28	0.28	6.36	16.93	-12.09
156	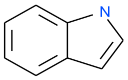	7-aza-bicyclo[4.3.0]nona-1,3,5,8-tetraene	C8H7N	117.058	-17.94	2.19	10.02	112.41	16	0	-2.17	-3.41	-13.73	0	3.41	1.53	-64.14
157	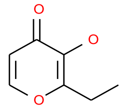	2-ethyl-3-hydroxy-4H-pyran-4-one	C7H8O3	140.047	-17.91	0.33	34.44	164.93	18	1	-5.08	-3.47	-14.8	1.4	12.16	3.55	-87.78
158	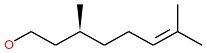	(S)-3,7-dimethyl-oct-6-en-1-ol	C10H20O	156.151	-17.9	3.51	17.17	199.03	31	6	-1.65	-4.95	-14.02	0	2.36	-2.68	-33.75
159	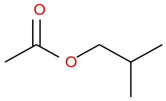	2-methyl-propyl ethanoate	C6H12O2	116.084	-17.86	1.52	21.74	129.46	20	2	-6.46	-3.41	-13.8	4.86	4.11	15.58	-7.91
160	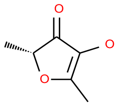	(R)-4-hydroxy-2,5-dimethyl-2,3-dihydro-furan-3-one	C6H8O3	128.047	-17.85	-0.21	36.87	140.39	17	0	-5.48	-3.2	-13.54	0.02	12.38	3.66	-86.8
161	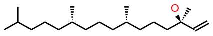	(3R,7S,11S)-3,7,11,15-tetramethyl-hexadec-1-en-3-ol	C20H40O	296.308	-17.84	8.2	16.84	380.16	61	14	0	-7.93	-20.04	8.69	-2.07	1.01	-134.15
162	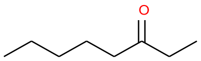	octan-3-one	C8H16O	128.12	-17.84	2.78	13.27	159.96	25	5	-3.03	-4.69	-17.88	0.54	7.11	3.8	-91.83
163	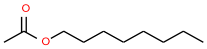	octyl ethanoate	C10H20O2	172.146	-17.83	3.61	21.66	204.15	32	7	-3.75	-5.66	-16.21	0.62	8.88	1.21	-79.57
164	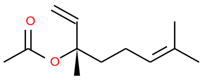	(S)-1,5-dimethyl-1-vinyl-hex-4-enyl ethanoate	C12H20O2	196.146	-17.81	3.54	20.24	264.72	34	5	0	-5.63	-16.23	0	1.66	-3.12	-45.31

165	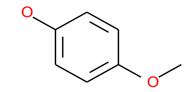	4-methoxy-phenol	C7H8O2	124.052	-17.78	1.69	25.4	118.29	17	0	-5.55	-3.53	-12.79	0.51	10.4	5.5	-80.39
166	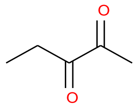	pentane-2,3-dione	C5H8O2	100.052	-17.78	0.05	27.58	113.02	15	2	-7	-2.42	-14.3	0	5.18	16.32	-16.45
167	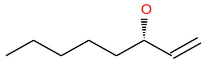	(S)-oct-1-en-3-ol	C8H16O	128.12	-17.75	2.81	17.13	157.78	25	6	-3.6	-4.65	-16.82	0.61	8.38	2.15	-75.72
168	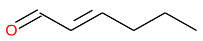	hex-2-enal	C6H10O	98.073	-17.72	1.94	14.61	126.21	17	2	-4.43	-3.13	-12.59	0.8	6.59	3.07	-52.65
169	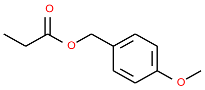	(4-methoxy-phenyl)-methyl propanoate	C11H14O3	194.094	-17.69	2.44	28.32	200	28	3	-3.54	-5.22	-18.01	3.11	9.02	6.38	-109.55
170	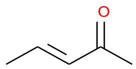	pent-3-en-2-one	C5H8O	84.058	-17.62	1.14	13.84	108.71	14	0	-2.9	-3.18	-11.93	0	3.95	1.25	-40.41
171	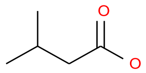	3-methyl-butanoic acid	C5H10O2	102.068	-17.62	1.19	28.89	105.34	16	2	-5.84	-2.41	-9.41	1.73	5.22	4.64	-13.7
172	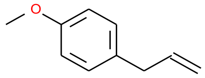	1-allyl-4-methoxy-benzene	C10H12O	148.089	-17.61	2.99	7.78	169.84	23	2	-2.7	-5.04	-16.25	0.51	6.72	3.94	-81.75
173	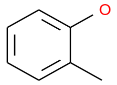	o-cresol	C7H8O	108.058	-17.61	1.88	16.79	107.59	16	0	-2.43	-3.37	-9.72	0	2.31	-1.83	-24.94
174	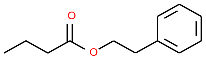	2-phenyl-ethyl butanoate	C12H16O2	192.115	-17.6	3.14	20.69	203.5	30	5	-3.99	-5.09	-12.44	3.6	6.66	0.52	-80.55
175	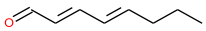	octa-2,4-dienal	C8H12O	124.089	-17.59	2.57	14.61	167.67	21	2	-2.59	-4.11	-17.66	0.7	7.7	3.64	-65.01
176	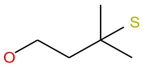	3-mercapto-3-methyl-butan-1-ol	C5H12OS	120.061	-17.56	1.35	17.17	130.24	19	4	-4.54	-2.85	-8.55	0.64	4.01	-1.56	-13.85
177	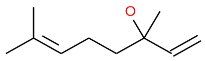	3,7-dimethyl-octa-1,6-dien-3-ol	C10H18O	154.136	-17.55	3.25	16.84	217.74	29	5	-1.56	-4.99	-14.54	0	2.78	-1.23	-23.14
178	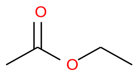	ethyl ethanoate	C4H8O2	88.052	-17.54	0.72	21.37	96.21	14	1	-3.79	-2.89	-8.94	0	4.21	-1.27	-1.68
179	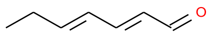	hepta-2,4-dienal	C7H10O	110.073	-17.51	2.09	14.61	149.18	18	1	-2.65	-3.63	-16.43	0.23	6.84	3.51	-56.63

180	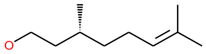	(R)-3,7-dimethyl-oct-6-en-1-ol	C10H20O	156.151	-17.5	3.51	17.17	199.03	31	6	-2.89	-4.53	-12.3	0	3.15	-1.22	-38.97
181	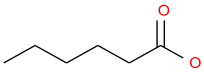	hexanoic acid	C6H12O2	116.084	-17.5	1.84	28.89	126.86	19	4	-6.44	-2.82	-4.82	4.27	5.22	-1.23	-15.18
182	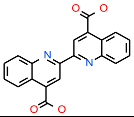	3-(3-carboxy-2-aza-bicyclo[4.4.0]deca-1(10),2,4,6,8-pentaen-3-yl)-2-aza-bicyclo[4.4.0]deca-1(10),2,4,6,8-pentaene-	C20H12N2O4	344.08	-17.49	3.94	73.44	306.35	36	2	-1.97	-4.72	-14.53	0	5.06	0.29	-71.67
183	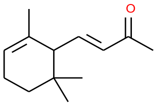	4-(2,6,6-trimethyl-cyclohex-2-enyl)-but-3-en-2-one	C13H20O	192.151	-17.48	4.05	13.77	264.15	34	1	0	-5.65	-13.73	0	0.23	-1.12	-36.65
184	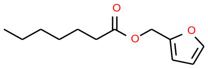	(furan-2-yl)-methyl heptanoate	C12H18O3	210.126	-17.46	3.44	28.52	224.58	33	7	0	-5.23	-18.67	2.39	3.17	-2.82	-47.24
185	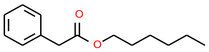	hexyl 2-phenyl-ethanoate	C14H20O2	220.146	-17.45	4.16	20.69	239.46	36	7	-3.11	-6.53	-19.67	1.46	9.92	4.28	-86.96
186	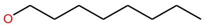	octan-1-ol	C8H18O	130.136	-17.39	3.04	17.17	158.09	27	7	-4.49	-4.64	-15.79	2.85	8.39	3.59	-84.62
187	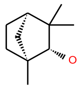	(1S,2R,4R)-1,3,3-trimethyl-bicyclo[2.2.1]heptan-2-ol	C10H18O	154.136	-17.38	2.75	16.59	198.5	29	1	-2.64	-4.18	-9.57	0	2.69	-1.42	-35.51
188	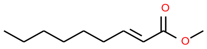	methyl non-2-enoate	C10H18O2	170.131	-17.37	3.77	21.09	212.18	30	5	-2.77	-5.52	-20.51	5.05	9.29	5.88	-92.01
189	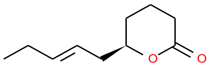	(R)-2-oxo-6-(pent-2-enyl)-tetrahydro-2H-pyran	C10H16O2	168.115	-17.35	2.16	21.06	199.52	28	3	-3.29	-5	-17.79	2.2	8.96	5.44	-99.02
190	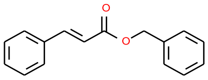	phenyl-methyl 3-phenyl-prop-2-enoate	C16H14O2	238.099	-17.34	4.29	20.5	247.8	32	3	0	-5.57	-17.58	1.6	1.42	1.68	-97.61
191	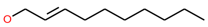	dec-2-en-1-ol	C10H20O	156.151	-17.34	3.7	17.17	198.98	31	8	-4.46	-5.39	-17.68	4.96	8.56	6.09	-93.87
192	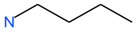	butan-1-amine	C4H11N	73.089	-17.34	0.51	21.67	88.53	16	3	-4.37	-2.81	-10.88	1.32	6.86	-0.7	-50.46
193	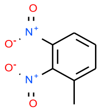	1-methyl-2,3-dinitro-benzene	C7H6N2O4	182.033	-17.33	1.47	75.86	149.26	19	2	0	-3.36	-13.6	0	0	-4.06	-34.51
194	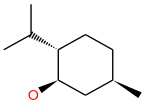	(1R,2S,5R)-2-isopropyl-5-methyl-cyclohexanol	C10H20O	156.151	-17.32	2.95	16.05	180.11	31	2	-2.18	-4.55	-11.26	0.43	2.51	-0.86	-39.28

195	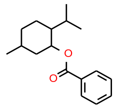	2-isopropyl-5-methyl-cyclohexyl benzoate	C17H24O2	260.178	-17.31	5.04	20.27	278.86	43	3	0	-6.3	-13.16	0.99	0.12	-2.74	-64.52
196	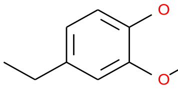	4-ethyl-2-methoxy-phenol	C9H12O2	152.084	-17.3	2.47	24.42	158.25	23	1	-4.47	-4.42	-15.14	0.39	10.12	5.94	-87.38
197	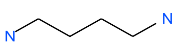	butane-1,4-diamine	C4H12N2	88.1	-17.3	-1.25	42.82	98.57	18	5	-6.61	-2.38	-11.25	1.26	11.46	1.02	-47.76
198	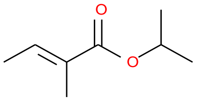	1-methyl-ethyl 2-methyl-but-2-enoate	C8H14O2	142.099	-17.28	2.33	20.94	173.55	24	1	0	-4.52	-13.36	0	0.02	-2.28	-35.84
199	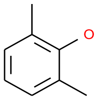	2,6-dimethyl-phenol	C8H10O	122.073	-17.28	2.16	15.72	126.7	19	0	-2.62	-3.75	-9.95	0	2.05	0.41	-30.63
200	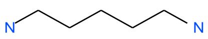	pentane-1,5-diamine	C5H14N2	102.116	-17.27	-0.76	42.82	116.48	21	6	-6.31	-2.69	-13.24	4.29	12	1.73	-49.69
201	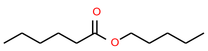	pentyl hexanoate	C11H22O2	186.162	-17.26	4.11	20.96	221.09	35	8	-2.52	-5.43	-21.72	3.14	9.07	4.37	-123.2
202	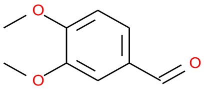	3,4-dimethoxy-benzaldehyde	C9H10O3	166.063	-17.26	1.71	29.66	167.35	22	0	0	-3.64	-17.47	0.09	2.93	0.7	-43.37
203	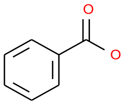	benzoic acid	C7H6O2	122.037	-17.23	1.56	28.65	109	14	1	-6.26	-2.35	-5.83	0.31	5.22	2.28	0.36
204	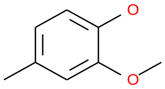	2-methoxy-4-methyl-phenol	C8H10O2	138.068	-17.19	1.97	24.42	140.11	20	0	-4.42	-4.08	-15.32	0	10.12	6.74	-83.98
205	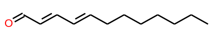	dodeca-2,4-dienal	C12H20O	180.151	-17.18	4.5	14.61	239.29	33	6	-1.69	-5.88	-23.07	0.69	8.38	6.41	-73.62
206	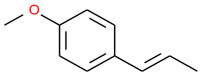	1-methoxy-4-(prop-1-enyl)-benzene	C10H12O	148.089	-17.18	3.29	7.78	167.14	23	0	0	-4.58	-13.78	0	0.59	-1.17	-30.8
207	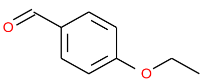	4-ethoxy-benzaldehyde	C9H10O2	150.068	-17.16	2.22	21.52	154.49	21	1	-2.82	-3.93	-17.88	0.3	8.87	4.93	-74.48
208	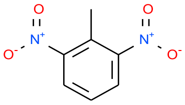	2-methyl-1,3-dinitro-benzene	C7H6N2O4	182.033	-17.16	1.47	76.16	150.2	19	2	0	-3.03	-14.3	0	0	-3.08	-39.5
209	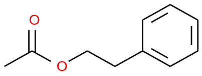	2-phenyl-ethyl ethanoate	C10H12O2	164.084	-17.14	2.17	21.39	168.65	24	3	0	-4.65	-16.53	0	1.04	-0.1	-39.96

210	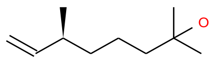	(S)-2,6-dimethyl-oct-7-en-2-ol	C10H20O	156.151	-17.12	3.45	16.7	202.43	31	6	-1.38	-5.12	-15.2	1.89	2.87	-1.14	-21.02
211	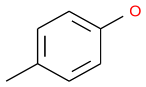	p-cresol	C7H8O	108.058	-17.1	2	17.86	107.38	16	0	-2.77	-2.87	-10.69	0	3.18	0.2	-31.33
212	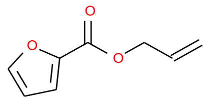	allyl furan-2-carboxylate	C8H8O3	152.047	-17.08	1.89	29.86	157	19	2	0	-4.09	-15.97	0.7	1.93	-1.53	-37.87
213	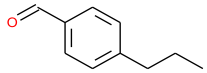	4-propyl-benzaldehyde	C10H12O	148.089	-17.06	3.03	14.4	160.7	23	2	-2.65	-4.11	-17.89	1.23	7.76	4.97	-89.07
214	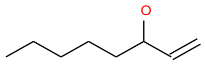	oct-1-en-3-ol	C8H16O	128.12	-17.03	2.81	17.13	157.78	25	6	-3.45	-4.64	-16.8	1.02	8.38	2.63	-76.39
215	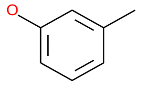	m-cresol	C7H8O	108.058	-16.99	2	17.86	107.46	16	0	-2.18	-3.01	-11.82	0	3.17	-0.02	-39.08
216	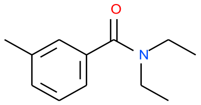	diethylamino-m-tolyl-methanone	C12H17NO	191.131	-16.96	2.7	16.17	216.2	31	3	0	-5.49	-14.84	0	1.39	-1.96	-52.1
217	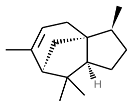	(3S,3aR,7R,8aS)-3,6,8,8-tetramethyl-2,3,4,7,8,8a-hexahydro-1H-3a,7-methanoazulene	C15H24	204.351	-16.91	5.28	0	276.98	39	0	0	-5.29	-12.52	0.01	0	-1.29	-50.83
218	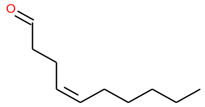	(Z)-dec-4-enal	C10H18O	154.136	-16.89	3.4	14.61	201.12	29	7	-2.82	-4.79	-19.87	0.39	7.96	4.76	-100.6
219	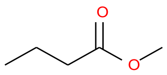	methyl butanoate	C5H10O2	102.068	-16.89	1.22	21.09	114.05	17	2	-3.91	-3.29	-8.1	0.32	4.42	-1.85	-6.58
220	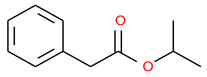	1-methyl-ethyl 2-phenyl-ethanoate	C11H14O2	178.099	-16.88	2.49	20.08	182.66	27	3	-3.07	-5.28	-19.24	2.52	8.8	8	-127.64
221	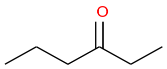	hexan-3-one	C6H12O	100.089	-16.87	1.82	13.27	124.15	19	3	-3.76	-3.69	-10.82	0	4.47	1.18	-55.66
222	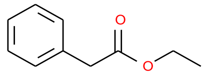	ethyl 2-phenyl-ethanoate	C10H12O2	164.084	-16.85	2.23	20.4	167.34	24	3	-3.95	-4.62	-9.57	1.57	5.12	0.26	-32.45
223	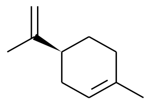	(S)-4-isopropenyl-1-methyl-cyclohex-1-ene	C10H16	136.125	-16.85	3.83	0	195.06	26	1	0	-4.77	-13.65	0	0	-0.91	-29.4
224	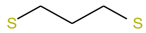	propane-1,3-dithiol	C3H8S2	108.007	-16.82	1.8	0	90.74	13	4	-4.35	-2.24	-8.22	0.05	3.29	-1.46	-15.78

225	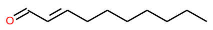	dec-2-enal	C10H18O	154.136	-16.8	3.87	14.61	197.83	29	6	-2.87	-4.84	-20.35	2.9	8.03	6.71	-88.69
226	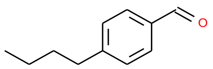	4-butyl-benzaldehyde	C11H14O	162.104	-16.8	3.51	14.4	178.6	26	3	-3.49	-4.53	-15.96	2.65	7.49	5.48	-73.27
227	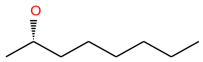	(S)-octan-2-ol	C8H18O	130.136	-16.79	3.03	16.99	155.57	27	6	-4.16	-4.73	-13.67	2.05	8.53	0.89	-94.04
228	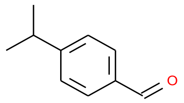	4-isopropyl-benzaldehyde	C10H12O	148.089	-16.78	2.77	14.4	157.14	23	1	-2.66	-4.11	-18.01	0.23	7.95	6.28	-82.93
229	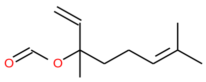	(1,5-dimethyl-1-vinyl-hex-4-enyloxy)-methanone	C11H18O2	182.131	-16.77	3.22	20.88	244.51	31	5	0	-5.16	-15.68	0	0.53	-1.57	-49.78
230	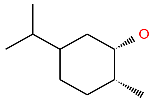	(1S,2R)-5-isopropyl-2-methyl-cyclohexanol	C10H20O	156.151	-16.77	2.95	16.05	179.36	31	2	-2.04	-4.22	-11.62	0.86	3.08	-1	-40.79
231	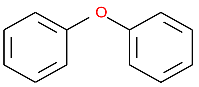	phenoxy-benzene	C12H10O	170.073	-16.75	3.3	6.99	165.46	23	2	0	-4.76	-15.12	0	1.22	-0.84	-51.55
232	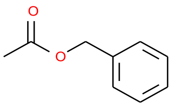	phenyl-methyl ethanoate	C9H10O2	150.068	-16.73	1.86	21.47	150.77	21	2	-2.9	-4.32	-17.67	0	8.04	5.98	-85.62
233	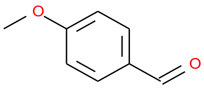	4-methoxy-benzaldehyde	C8H8O2	136.052	-16.71	1.74	21.94	135.92	18	0	-2.64	-3.48	-16.92	0	9.15	3.59	-73.97
234	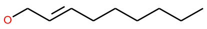	non-2-en-1-ol	C9H18O	142.136	-16.7	3.22	17.17	181.08	28	7	-2.29	-4.83	-14.18	2.58	3.27	-0.21	-15.68
235	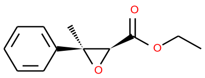	(2S,3S)-ethyl 3-methyl-3-phenyl-oxirane-2-carboxylate	C12H14O3	206.094	-16.69	2.59	28.74	221.12	29	3	-3.15	-5.01	-18.18	0.82	10.71	4.5	-121.56
236	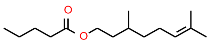	3,7-dimethyl-oct-6-enyl pentanoate	C15H28O2	240.209	-16.69	5.54	20.96	297.84	45	9	-3.6	-6.53	-17.09	4.05	9.29	2.32	-88.68
237	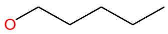	pentan-1-ol	C5H12O	88.089	-16.69	1.59	17.17	104.37	18	4	-3.67	-3.25	-11.32	0.67	5.52	-0.66	-53.93
238	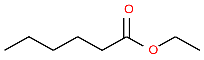	ethyl hexanoate	C8H16O2	144.115	-16.67	2.66	20.67	166.87	26	5	-3.26	-4.86	-17.06	0	8.51	3.81	-84.94
239	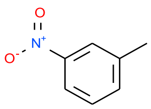	1-methyl-3-nitro-benzene	C7H7NO2	137.048	-16.67	1.99	38.5	122.54	17	1	0	-3.15	-14.37	0	0	-1.19	-38.84

240	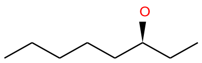	(S)-octan-3-ol	C8H18O	130.136	-16.66	3.12	17.13	155.4	27	6	-4.49	-4.88	-16.68	2.71	9.03	6.48	-91.98
241	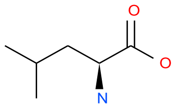	(S)-2-amino-4-methyl-pentanoic acid	C6H13NO2	132.102	-16.65	-0.47	49.71	133.58	23	5	-6.72	-2.73	-16.52	0.64	13.44	8.53	-72.29
242	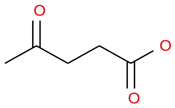	4-oxo-pentanoic acid	C5H8O3	117.055	-16.65	-0.08	42.23	117.6	17	4	-6.54	-2.4	-15.6	0	8.95	12.12	-61.52
243	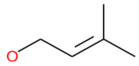	3-methyl-but-2-en-1-ol	C5H10O	86.073	-16.65	1.33	17.17	110.67	16	2	-4.03	-3.21	-10.82	0	6.57	0.59	-52.2
244	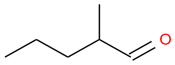	2-methyl-pentanal	C6H12O	100.089	-16.63	1.78	14.67	117.26	19	3	-3.94	-3.25	-10.47	0.65	5.18	0.45	-56.18
245	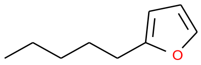	2-pentyl-furan	C9H14O	138.104	-16.6	3.36	7.99	153.57	24	4	-2.85	-4.94	-18.22	0.97	7.45	6.28	-95.05
246	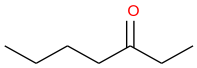	heptan-3-one	C7H14O	114.104	-16.6	2.3	13.27	142.06	22	4	-2.92	-4.08	-13.78	0.75	4.65	2.12	-58.53
247	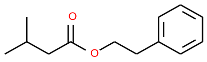	2-phenyl-ethyl 3-methyl-butanoate	C13H18O2	206.131	-16.6	3.47	20.69	217.78	33	5	0	-5.22	-16.15	1.15	1.38	-1.5	-47.05
248	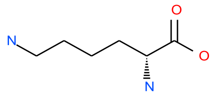	(R)-2,6-diamino-hexanoic acid	C6H14N2O2	147.113	-16.59	-2.07	70.86	144.8	25	8	-9.98	-2.44	-19.98	0.9	20.95	14.78	-81.8
249	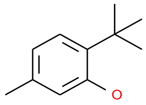	5-methyl-2-tert-butyl-phenol	C11H16O	164.12	-16.59	3.49	16.79	193.7	28	1	-2.25	-4.89	-10.15	0.75	2.31	0.28	-41.13
250	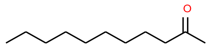	undecan-2-one	C11H22O	170.167	-16.58	4.22	13.84	212.47	34	8	-3.8	-5.9	-18.64	5.54	7.96	7.39	-105.37
251	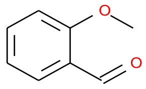	2-methoxy-benzaldehyde	C8H8O2	136.052	-16.58	1.62	22.03	134.6	18	0	-3.65	-3.55	-14.54	0.48	8.65	4.67	-82.43
252	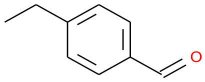	4-ethyl-benzaldehyde	C9H10O	134.073	-16.58	2.55	14.4	143.16	20	1	0	-3.14	-16.53	0.18	1.77	0.2	-44.05
253	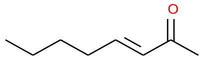	oct-3-en-2-one	C8H14O	126.104	-16.57	2.5	13.84	166.04	23	3	-2.86	-4.52	-16.49	0	6.9	4.85	-71.06
254	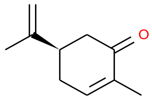	(R)-5-isopropenyl-2-methyl-cyclohex-2-enone	C10H14O	150.104	-16.55	2.52	13.68	206.18	25	1	0	-4.63	-13.88	0.2	0.97	-1.35	-36.08

255	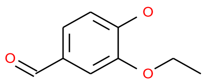	3-ethoxy-4-hydroxy-benzaldehyde	C9H10O3	166.063	-16.54	1.84	38.16	165.91	22	1	0	-3.51	-17.12	0.52	2.11	1.14	-45.74
256	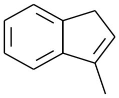	7-methyl-bicyclo[4.3.0]nona-1,3,5,7-tetraene	C10H10	130.078	-16.53	3.48	0	157.79	20	0	0	-4.21	-12.75	0	0	-1.46	-41.64
257	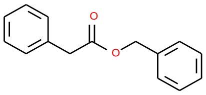	phenyl-methyl 2-phenyl-ethanoate	C15H14O2	226.099	-16.52	3.37	20.5	221.9	31	4	0	-5.24	-17.64	0.21	1.22	1.98	-95.18
258	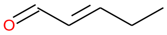	pent-2-enal	C5H8O	84.058	-16.51	1.46	14.61	107.72	14	1	-2.68	-2.67	-12.41	0.27	3.78	1.54	-54.7
259	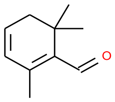	2,6,6-trimethyl-cyclohexa-1,3-dienecarbaldehyde	C10H14O	150.104	-16.5	2.72	14.59	216.78	25	0	0	-3.97	-15.15	0.55	1.7	0.05	-36.26
260	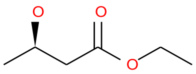	(R)-ethyl 3-hydroxy-butanoate	C6H12O3	132.079	-16.5	0.53	37.15	135.59	21	4	-2.54	-3.39	-12.28	0	4.54	-2.01	-28.91
261	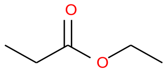	ethyl propanoate	C5H10O2	102.068	-16.48	1.22	20.67	113.59	17	2	-3.61	-3.4	-9.82	2.24	4.24	0.55	-20.03
262	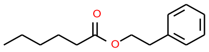	2-phenyl-ethyl hexanoate	C14H20O2	220.146	-16.46	4.11	20.69	239.31	36	7	-2.95	-5.69	-19.01	6.25	8.65	4.74	-112.76
263	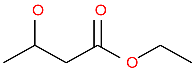	ethyl 3-hydroxy-butanoate	C6H12O3	132.079	-16.44	0.53	37.15	135.59	21	4	-8.68	-3.17	-9.91	5.11	7.41	14.54	-0.29
264	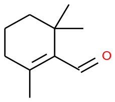	2,6,6-trimethyl-cyclohex-1-enecarbaldehyde	C10H16O	152.12	-16.42	3.06	14.59	210.5	27	0	-4.2	-4.3	-10.87	1.18	7.37	3.68	-98.24
265	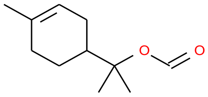	(1-methyl-1-(4-methyl-cyclohex-4-enyl)-ethoxy)-methanone	C11H18O2	182.131	-16.4	2.93	21.02	228.59	31	2	0	-4.64	-14.77	2.44	1.27	-1.02	-44.35
266	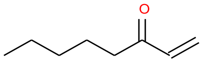	oct-1-en-3-one	C8H14O	126.104	-16.38	2.6	13.27	163.66	23	4	-2.49	-4.76	-17.62	0.86	7.41	4.2	-84.37
267	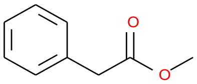	methyl 2-phenyl-ethanoate	C9H10O2	150.068	-16.37	1.75	20.82	150.33	21	2	-3.24	-4.22	-12.4	0.55	7.06	0.85	-62.75
268	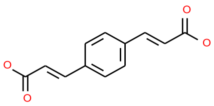	3-(4-(2-carboxy-vinyl)-phenyl)-acrylic acid	C12H10O4	218.058	-16.36	2.8	57.01	227.31	24	2	-1.91	-3.42	-14.23	0	6.16	-1.27	-42.96
269	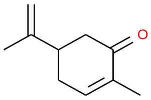	5-isopropenyl-2-methyl-cyclohex-2-enone	C10H14O	150.104	-16.35	2.52	13.68	206.18	25	1	0	-4.61	-14.35	0	1.01	-0.42	-31.04

270	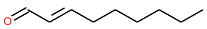	non-2-enal	C9H16O	140.12	-16.34	3.38	14.61	179.93	26	5	-2.9	-4.5	-18.95	2.13	7.95	6.04	-86.55
271	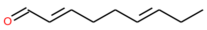	nona-2,6-dienal	C9H14O	138.104	-16.34	2.92	14.61	186.2	24	4	0	-4	-17.77	0.82	1.79	0.54	-33.66
272	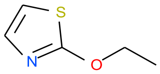	2-ethoxy-thiazole	C5H7NOS	129.025	-16.34	1.62	19.59	112.23	15	1	0	-3.76	-13.76	0.17	1.71	-2.94	-26.64
273	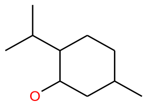	2-isopropyl-5-methyl-cyclohexanol	C10H20O	156.151	-16.31	2.95	16.05	180.11	31	2	-1.84	-4.59	-11.24	0.21	2.74	-0.91	-37.61
274	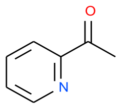	1-(pyridin-2-yl)-ethanone	C7H7NO	121.053	-16.3	0.67	23.11	117.02	16	0	-6.6	-2.94	-10.92	0.02	4.68	14.81	3.17
275	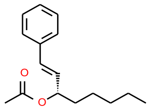	(S)-1-styryl-hexyl ethanoate	C16H22O2	246.162	-16.28	4.98	20.54	278.51	40	7	0	-6.45	-19.19	0	1.45	2.94	-108.71
276	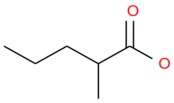	2-methyl-pentanoic acid	C6H12O2	116.084	-16.26	1.67	28.92	124.09	19	3	-5.45	-2.86	-7.23	1.22	5.2	1.46	-16.43
277	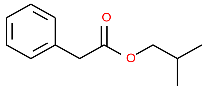	2-methyl-propyl 2-phenyl-ethanoate	C12H16O2	192.115	-16.25	3.03	20.77	200.59	30	4	0	-5.56	-14.9	0.58	1.95	-2.26	-52.48
278	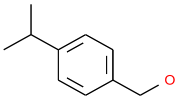	(4-isopropyl-phenyl)-methanol	C10H14O	150.104	-16.24	2.4	17.35	157.12	25	3	-3.79	-4.71	-15.33	0.53	9.38	4.56	-90.89
279	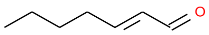	hept-2-enal	C7H12O	112.089	-16.24	2.42	14.61	144.11	20	3	-2.86	-3.7	-16.53	0	7.09	4.4	-80.91
280	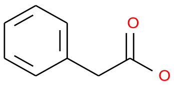	2-phenyl-acetic acid	C8H8O2	137.06	-16.24	1.4	28.62	127.33	19	3	-2.36	-3.13	-14.19	0.97	3.76	2.12	-47.96
281	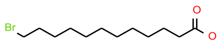	12-bromo-dodecanoic acid	C12H23BrO2	278.088	-16.14	5.05	28.89	253.44	37	11	-6.84	-6.26	-10.81	9.58	10.36	2.73	-105.95
282	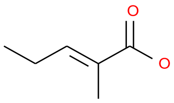	2-methyl-pent-2-enoic acid	C6H10O2	114.068	-16.07	1.64	28.92	136.54	17	2	-6.38	-2.74	-4.46	0	5.24	1.86	-4.97
283	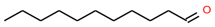	undecanal	C11H22O	170.167	-16.06	4.35	14.61	212.17	34	9	-2.81	-5.43	-21.14	6.29	8.02	6.69	-118.36
284	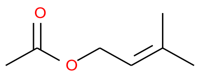	3-methyl-but-2-enyl ethanoate	C7H12O2	128.084	-16.05	1.91	21.66	158.33	21	2	0	-4.3	-13.64	0	1.32	-2.59	-30.7

285	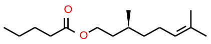	(S)-3,7-dimethyl-oct-6-enyl pentanoate	C15H28O2	240.209	-16.04	5.54	20.96	297.84	45	9	-3.59	-6.55	-15.34	0	9.31	0.6	-76.09
286	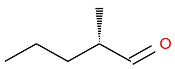	(S)-2-methyl-pentanal	C6H12O	100.089	-16.02	1.78	14.67	117.26	19	3	-4.49	-3.39	-13.88	1.39	7.47	6.21	-73.84
287	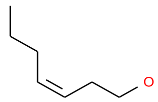	(Z)-hept-3-en-1-ol	C7H14O	114.104	-16	2.09	17.17	147.03	22	5	-2.23	-3.74	-12.62	0.07	3.2	-0.98	-11.03
288	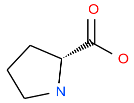	(R)-pyrrolidine-2-carboxylic acid	C5H9NO2	116.071	-15.98	-0.82	41.32	113.22	18	2	-4.83	-2.41	-13.12	2.07	8.53	5.09	-65.28
289	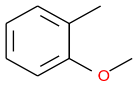	1-methoxy-2-methyl-benzene	C8H10O	122.073	-15.98	2.23	7.87	127.59	19	0	0	-3.98	-12.76	0.05	0.57	-1.48	-42.25
290	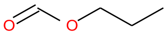	propoxy-methanone	C4H8O2	88.052	-15.97	0.88	22.76	93.44	14	2	-5.09	-2.33	-6.62	1.3	4.29	1.03	-13.94
291	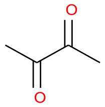	biacetyl	C4H6O2	86.037	-15.95	-0.45	28.16	95.8	12	1	-7.43	-2	-10.75	0	5.23	15.78	-11.08
292	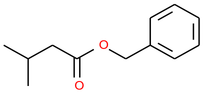	phenyl-methyl 3-methyl-butanoate	C12H16O2	192.115	-15.92	3.16	20.77	199.91	30	4	0	-5.37	-15.49	0.24	2.04	-1.14	-61.83
293	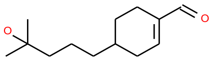	4-(4-hydroxy-4-methyl-pentyl)-cyclohex-1-enecarbaldehyde	C13H22O2	210.162	-15.9	3.69	30.78	268.82	37	5	-6.58	-5.11	-7.8	3.45	11.01	1.08	-117.04
294	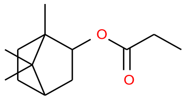	1,7,7-trimethyl-bicyclo[2.2.1]heptan-2-yl propanoate	C13H22O2	210.162	-15.89	3.69	20.59	262.08	37	2	0	-4.98	-14.62	1.4	1.34	-0.2	-49.41
295	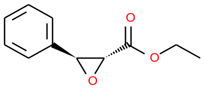	(2R,3S)-ethyl 3-phenyl-oxirane-2-carboxylate	C11H12O3	192.079	-15.88	2.15	29.44	192.59	26	3	-3.35	-4.78	-13.63	0.66	7.02	3.57	-54.11
296	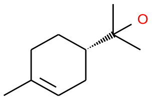	(S)-2-(4-methyl-cyclohex-3-enyl)-propan-2-ol	C10H18O	154.136	-15.88	2.95	16.15	205.6	29	2	0	-4.46	-12.22	0	-0.24	-2.56	-42.74
297	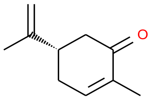	(S)-5-isopropenyl-2-methyl-cyclohex-2-enone	C10H14O	150.104	-15.86	2.52	13.68	206.18	25	1	0	-4.21	-12.44	0.27	1.32	-3.31	-27.72
298	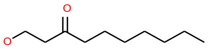	1-hydroxy-decan-3-one	C10H20O2	172.146	-15.8	2.59	29.92	203.32	32	9	-2.45	-5.17	-12.27	0	2.98	-2.26	-41.06
299	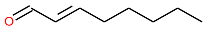	oct-2-enal	C8H14O	126.104	-15.79	2.9	14.61	162.02	23	4	-2.5	-4.13	-15.85	1.22	6.97	2.32	-77

300	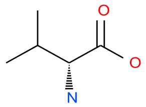	(R)-2-amino-3-methyl-butanoic acid	C5H11NO2	118.087	-15.78	-1.02	49.89	117.73	20	4	-7.38	-2.35	-11.46	1.45	13.66	4.97	-58.37
301	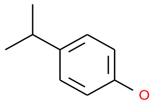	4-isopropyl-phenol	C9H12O	136.089	-15.76	2.72	17.86	139.51	22	1	-2.77	-3.46	-11.27	0.17	3.17	2.82	-52.99
302	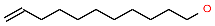	undec-10-en-1-ol	C11H22O	170.167	-15.75	4.1	17.17	215.65	34	10	-2.97	-5.92	-20.92	6.29	9.55	5.29	-102.31
303	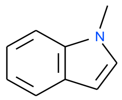	7-methyl-7-aza-bicyclo[4.3.0]nona-1(6),2,4,8-tetraene	C9H9N	131.073	-15.73	2.13	2.9	135.49	19	0	0	-4.03	-12.68	0.03	0	-0.54	-43.99
304	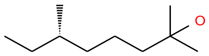	(S)-2,6-dimethyl-octan-2-ol	C10H22O	158.167	-15.73	3.93	16.7	200.24	33	6	-2.67	-4.6	-10.45	0.02	2.96	-1.91	-27.46
305	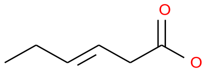	hex-3-enoic acid	C6H10O2	114.068	-15.72	1.42	28.89	131.75	17	3	-5.56	-2.76	-3.2	0.7	5.25	-3.57	-19.06
306	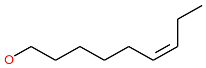	(Z)-non-6-en-1-ol	C9H18O	142.136	-15.71	3.05	17.17	182.26	28	7	-3.4	-4.79	-18.54	0.43	9.52	4.99	-95.82
307	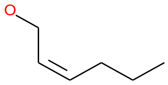	(Z)-hex-2-en-1-ol	C6H12O	100.089	-15.71	1.78	17.17	127.36	19	4	-2.73	-3.45	-11.63	0	3.28	0.42	-25.16
308	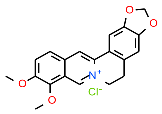	9,10-Dimethoxy-5,6-dihydro[1,3]dioxolo[4,5-g]isoquinolino[3,2-a]isoquinolin-7-ium chloride	C20H18ClNO4	371.814	-15.64	5.38	33.45	340.03	43	0	0	-5.97	-15.45	0	2.64	2.78	-115.84
309	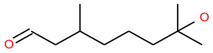	7-hydroxy-3,7-dimethyl-octanal	C10H20O2	172.146	-15.63	2.63	30.8	207.15	32	7	-5.23	-4.38	-13.86	3.46	10.08	4	-92.45
310	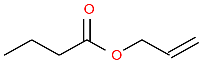	allyl butanoate	C7H12O2	128.084	-15.63	1.97	20.96	153.14	21	4	-3.08	-4.37	-18.23	0	8.75	6.58	-87.73
311	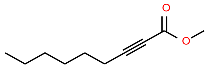	methyl non-2-ynoate	C10H16O2	168.115	-15.63	2.97	21.09	209.95	28	5	-2.15	-5.38	-20.95	2.18	8.62	7.43	-59.32
312	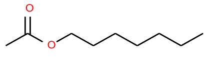	heptyl ethanoate	C9H18O2	158.131	-15.6	3.13	21.66	186.24	29	6	-2.94	-5.16	-19.12	2.25	8.96	6.21	-95.79
313	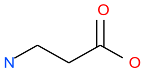	3-amino-propanoic acid	C3H7NO2	90.056	-15.59	-1.37	50.05	83.19	14	4	-7.14	-1.5	-10.6	0	12.25	3.77	-43.49
314	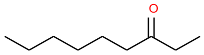	nonan-3-one	C9H18O	142.136	-15.58	3.27	13.27	177.87	28	6	-2.91	-5.02	-16.38	1.67	7.1	3.92	-85.06

315	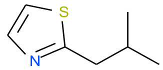	2-isobutyl-thiazole	C7H11NS	141.061	-15.58	2.31	11.17	135.59	20	2	0	-4.32	-12.56	0.52	0.49	-2.55	-35.48
316	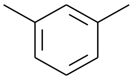	m-xylene	C8H10	106.078	-15.51	2.66	0	117.85	18	0	0	-3.84	-12.07	0.05	0	-1.32	-40.32
317	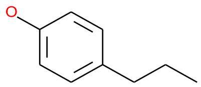	4-propyl-phenol	C9H12O	136.089	-15.5	2.98	17.86	143.06	22	2	-3.08	-4.23	-16.83	0.94	8.66	6.46	-81.42
318	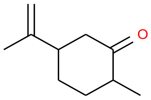	5-isopropenyl-2-methyl-cyclohexanone	C10H16O	152.12	-15.5	2.58	13.68	188.23	27	1	-4.11	-4.7	-10.09	0.71	7.44	2.98	-70.01
319	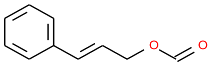	(3-phenyl-allyloxy)-methanone	C10H10O2	162.068	-15.49	2.38	22.48	171.61	22	3	-2.57	-4.08	-18.73	0	8.86	6.11	-80.41
320	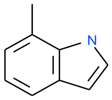	5-methyl-7-aza-bicyclo[4.3.0]nona-1(6),2,4,8-tetraene	C9H9N	131.073	-15.49	2.48	10.79	132.9	19	0	-2.27	-3.85	-11.17	0.13	3.34	2.25	-75.44
321	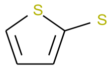	thiophene-2-thiol	C4H4S2	115.975	-15.47	2.05	0	86.55	10	1	-2.08	-3.51	-12.63	0	3.67	2.18	-43.05
322	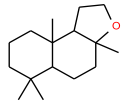	3a,6,6,9a-tetramethyl-2,4,5,5a,7,8,9,9b-octahydro-1H-benzo[e][1]benzofuran	C16H28O	236.393	-15.44	4.88	8.19	308.69	45	0	0	-5.58	-11.07	0.41	0.46	-1.47	-69.97
323	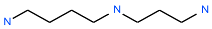	N1-(3-amino-propyl)-butane-1,4-diamine	C7H19N3	145.158	-15.44	-1.38	54.54	165.62	29	9	-6.25	-3.97	-17.87	1.63	17.55	3.55	-69.32
324	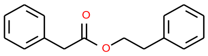	2-phenyl-ethyl 2-phenyl-ethanoate	C16H16O2	240.115	-15.43	3.68	20.42	239.78	34	5	-3.07	-6.27	-18.79	2.99	8.42	9.13	-127.33
325	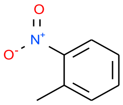	1-methyl-2-nitro-benzene	C7H7NO2	137.048	-15.43	1.87	38.2	122.16	17	1	0	-3.23	-11.52	0	0	-3.56	-22.29
326	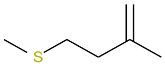	2-methyl-4-methylsulfanyl-but-1-ene	C6H12S	116.066	-15.42	2.43	0	138.56	19	3	0	-4.27	-12.96	0	0	-2.11	-34.5
327	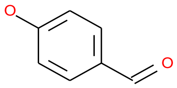	4-hydroxy-benzaldehyde	C7H6O2	122.037	-15.41	1.39	32.01	114.63	15	0	-1.89	-2.42	-11.64	0.63	3.51	0	-35.66
328	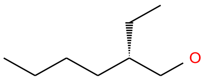	(S)-2-ethyl-hexan-1-ol	C8H18O	130.136	-15.38	3.07	17.63	155.72	27	6	-3.08	-4.08	-11.91	2.9	3.2	1.61	-57.86
329	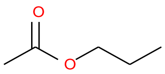	propyl ethanoate	C5H10O2	102.068	-15.38	1.2	21.66	114.61	17	2	-6.62	-3.07	-12.29	3.22	4.56	16.83	-4.67

330	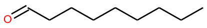	nonanal	C9H18O	142.136	-15.34	3.39	14.61	176.36	28	7	-3.34	-4.64	-17.07	2.01	7.08	5.71	-87.3
331	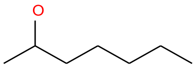	heptan-2-ol	C7H16O	116.12	-15.33	2.55	16.99	137.66	24	5	-4.31	-4.42	-14.26	3.28	8.43	5.27	-87.8
332	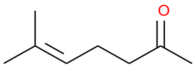	6-methyl-hept-5-en-2-one	C8H14O	126.104	-15.33	2.34	13.84	166.82	23	3	-2.92	-4.64	-16.55	0	7.81	6.14	-81.53
333	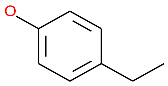	4-ethyl-phenol	C8H10O	122.073	-15.29	2.5	17.86	125.52	19	1	-2.97	-3.69	-14.79	0.42	8.02	4.34	-67.26
334	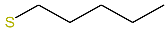	pentane-1-thiol	C5H12S	104.066	-15.28	2.76	0	111.47	18	4	-2.59	-3.17	-9.26	1.44	1.64	-1.46	-27.54
335	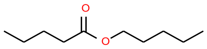	pentyl pentanoate	C10H20O2	172.146	-15.25	3.63	20.96	203.18	32	7	-2.53	-5.25	-21.84	3.22	9.09	8.3	-118.7
336	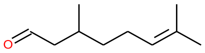	3,7-dimethyl-oct-6-enal	C10H18O	154.136	-15.25	3.37	14.61	198.02	29	5	-2.32	-4.79	-18.57	0	7.9	5.27	-97.48
337	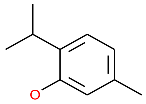	2-isopropyl-5-methyl-phenol	C10H14O	150.104	-15.25	3	16.79	162.81	25	1	-1.03	-4.45	-12.43	0	2.99	-0.13	-39.55
338	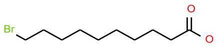	10-bromo-decanoic acid	C10H19BrO2	250.057	-15.21	4.09	28.89	217.63	31	9	-3.17	-5.48	-19.41	5.05	9.54	5.05	-99.09
339	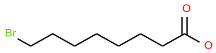	8-bromo-octanoic acid	C8H15BrO2	222.026	-15.19	3.12	28.89	181.81	25	7	-1.89	-3.71	-13.49	0.67	1.99	-0.2	-41.45
340	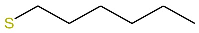	hexane-1-thiol	C6H14S	118.082	-15.18	3.24	0	129.38	21	5	-2.07	-3.69	-10.5	1.4	1.64	-1.84	-31.68
341	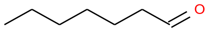	heptanal	C7H14O	114.104	-15.16	2.42	14.61	140.55	22	5	-2.88	-3.79	-16.43	1.39	6.98	4.33	-85.11
342	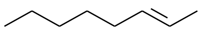	oct-2-ene	C8H16	112.125	-15.15	3.81	0	153.34	24	4	0	-4.73	-13.68	0	0	-1.05	-29.58
343	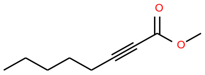	methyl oct-2-ynoate	C9H14O2	154.099	-15.14	2.49	21.09	192.05	25	4	-2.08	-4.86	-18.61	0.5	6.74	6.86	-48.81
344	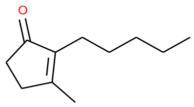	3-methyl-2-pentyl-cyclopent-2-enone	C11H18O	166.136	-15.14	3.34	14.04	223.77	30	4	0	-4.89	-12.39	0.94	0.46	-3.36	-45.1

345	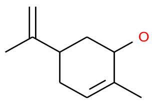	5-isopropenyl-2-methyl-cyclohex-2-enol	C10H16O	152.12	-15.12	2.81	16.05	200.94	27	2	-2.49	-4.08	-11.64	0	3.11	3.07	-53.84
346	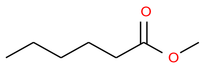	methyl hexanoate	C7H14O2	130.099	-15.11	2.18	21.09	149.87	23	4	-2.73	-4.47	-17.05	0.05	7.7	5.57	-83.56
347	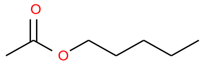	pentyl ethanoate	C7H14O2	130.099	-15.08	2.17	21.66	150.43	23	4	-4.06	-4.11	-6.6	2.28	4.25	-1.57	-2.82
348	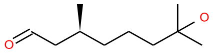	(S)-7-hydroxy-3,7-dimethyl-octanal	C10H20O2	172.146	-15.03	2.63	30.8	207.15	32	7	-4.87	-4.46	-14.6	4.11	10.46	4.31	-104.51
349	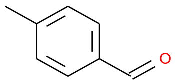	4-methyl-benzaldehyde	C8H8O	120.058	-14.99	2.05	14.4	125.02	17	0	0	-2.77	-14.74	0	1.76	0.42	-36.08
350	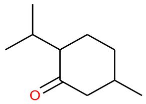	2-isopropyl-5-methyl-cyclohexanone	C10H18O	154.136	-14.95	2.74	13.68	182.71	29	1	-3.19	-4.84	-13.28	0.44	7.87	4.77	-104.13
351	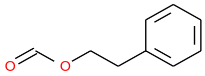	(2-phenyl-ethoxy)-methanone	C9H10O2	150.068	-14.95	1.85	22.48	147.48	21	3	0	-3.76	-15.09	0.79	0.82	0.38	-45.9
352	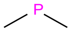	dimethylphosphane	C2H7P	62.0507	-14.93	0.71	0	49.28	10	0	-2.94	-2.36	-8.44	0.01	1.68	1.94	-17.89
353	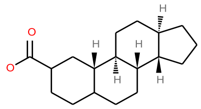	(8S,9R,10S,13R,14S)-hexadecahydro-1H-cyclopenta[a]phenanthrene-2-carboxylic acid	C18H28O2	276.414	-14.9	4.2	28.97	297.82	47	1	0	-5.73	-11.37	0	1.31	-1.93	-69.85
354	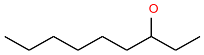	nonan-3-ol	C9H20O	144.151	-14.87	3.6	17.13	173.31	30	7	-2.02	-4.89	-12.51	3.39	2.98	-0.61	-36.33
355	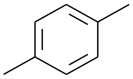	p-xylene	C8H10	106.078	-14.86	2.66	0	117.78	18	0	0	-3.77	-11.46	0.01	0	-1.33	-31.86
356	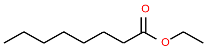	ethyl octanoate	C10H20O2	172.146	-14.82	3.63	20.67	202.69	32	7	-2.84	-5.65	-17.49	3.43	8.57	4.58	-95.93
357	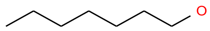	heptan-1-ol	C7H16O	116.12	-14.81	2.56	17.17	140.18	24	6	-2.07	-4.06	-12.2	2.2	3.26	-1.08	-22.45
358	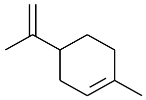	4-isopropenyl-1-methyl-cyclohex-1-ene	C10H16	136.125	-14.76	3.83	0	195.06	26	1	0	-4.06	-13.75	0.36	0.64	0.94	-25.18
359	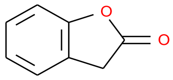	8-oxo-7-oxa-bicyclo[4.3.0]nona-1(6),2,4-triene	C8H6O2	134.037	-14.73	1.6	21.01	131.46	16	0	0	-3.2	-12.12	0	1.39	-2.29	-43.14

360	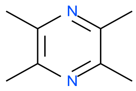	2,3,5,6-tetramethyl-pyrazine	C8H12N2	136.1	-14.69	1.05	19.78	149.71	22	0	0	-4.32	-12.26	0	2.06	-1.64	-27.31
361	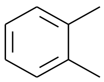	o-xylene	C8H10	106.078	-14.68	2.54	0	116.1	18	0	0	-3.73	-11.63	0	0	-0.83	-40.23
362	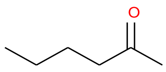	hexan-2-one	C6H12O	100.089	-14.66	1.81	13.84	122.94	19	3	-2.87	-3.69	-11.89	0	4.96	2.19	-51.1
363	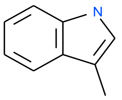	9-methyl-7-aza-bicyclo[4.3.0]nona-1(6),2,4,8-tetraene	C9H9N	131.073	-14.62	2.48	10.11	135.36	19	0	0	-3.88	-12.57	0.13	0.04	0.79	-47.53
364	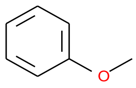	anisole	C7H8O	108.058	-14.55	1.95	7.78	107.74	16	1	-2.07	-3.65	-12.7	0	4.56	2.75	-70.23
365	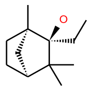	(1R,2R,4S)-2-ethyl-1,3,3-trimethyl-bicyclo[2.2.1]heptan-2-ol	C12H22O	182.167	-14.55	3.79	16.44	245.86	35	2	-1.82	-4.8	-9.41	0.18	2.44	-0.53	-37.63
366	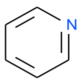	pyridine	C5H5N	79.042	-14.52	0.81	9.67	71.19	11	0	-2.85	-2.73	-8.82	0	3.54	1.06	-39.75
367	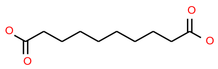	decanedioic acid	C10H18O4	202.121	-14.47	2.36	57.28	211.06	30	9	-7.22	-4.18	-7.97	4.43	13.79	-1.78	-62.4
368	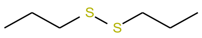	1-(propyldisulfanyl)propane	C6H14S2	150.305	-14.42	3.33	0	151.15	22	5	0	-4.95	-12.67	0.25	0	-2.17	-35.07
369	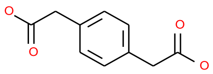	2-(4-(carboxy-methyl)-phenyl)-acetic acid	C10H10O4	194.058	-14.34	0.95	57.01	178.76	22	4	0	-3.95	-12.5	0.68	2.77	-5.57	-35.92
370	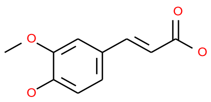	3-(4-hydroxy-3-methoxy-phenyl)-acrylic acid	C10H10O4	195.066	-14.31	2.04	52.8	194.87	25	1	-3.29	-3.72	-17.59	1.61	6.94	12.57	-32.56
371	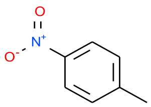	1-methyl-4-nitro-benzene	C7H7NO2	137.048	-14.31	1.99	38.5	122.47	17	1	0	-3.4	-11.75	0	0	-1.34	-30.34
372	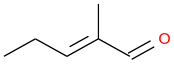	2-methyl-pent-2-enal	C6H10O	98.073	-14.28	1.73	14.67	127.14	17	1	0	-2.93	-14.65	0	1.76	0.61	-27.02
373	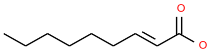	non-2-enoic acid	C9H16O2	156.115	-14.24	3.42	28.89	187.56	26	6	-1.93	-4.09	-10.98	0.78	3.9	-3.27	-53.15
374	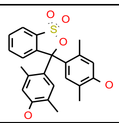	3,3-bis(4-hydroxy-2,5-dimethylphenyl)-3H-benzo[c][1,2]oxathiole 1,1-dioxide	C23H22O5S	410.483	-14.22	5.39	67.78	393.46	51	2	0	-6.42	-13.47	0	2.34	0.9	-79.85

375	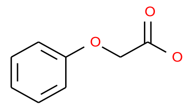	2-phenoxy-acetic acid	C8H8O3	153.055	-14.2	1.2	36.12	138.34	20	4	-3.13	-3.05	-15.14	2.23	5.46	6.68	-48.29
376	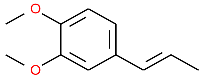	1,2-dimethoxy-4-(prop-1-enyl)-benzene	C11H14O2	178.099	-14.18	3.26	15.5	198.56	27	0	0	-4.72	-12.92	0	1.39	1.31	-71.4
377	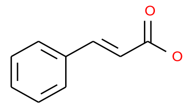	cinnamic acid	C9H8O2	149.06	-14.17	2.33	28.62	151.6	20	2	-2.29	-3.67	-14.58	0.82	4.89	5.75	-25.44
378	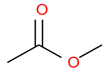	methyl ethanoate	C3H6O2	74.037	-14.15	0.24	21.79	79.2	11	0	-6.28	-2.29	-11.08	0	4.86	16.24	-14.97
379	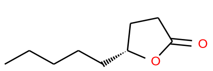	(R)-2-oxo-5-pentyl-tetrahydro-furan	C9H16O2	156.115	-14.12	2.27	21.43	177.62	27	4	0	-4.8	-13.4	1.95	1.48	-1.54	-43.57
380	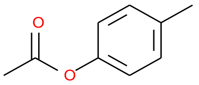	4-methyl-phenyl ethanoate	C9H10O2	150.068	-14.07	2	20.99	156.54	21	0	-1.37	-4.38	-18.02	0	8.3	6.25	-91.34
381	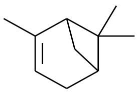	2,6,6-trimethyl-bicyclo[3.1.1]hept-2-ene	C10H16	136.125	-14.06	3.89	0	200.77	26	0	0	-4.13	-10.46	0.1	0	-1.26	-35.74
382	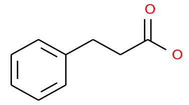	3-phenyl-propanoic acid	C9H10O2	150.068	-14.06	1.84	28.62	145.08	20	3	-2.05	-3.02	-9.85	0.76	4.14	-2.89	-34.92
383	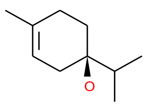	(R)-1-isopropyl-4-methyl-cyclohex-3-enol	C10H18O	154.136	-14.06	2.96	15.54	204.53	29	2	0	-4.6	-11.67	0.13	0.2	-0.99	-34.01
384	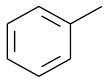	toluene	C7H8	92.063	-14.05	2.26	0	96.84	15	0	0	-3.38	-10.76	0.02	0	-1.57	-33.79
385	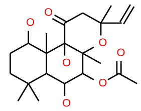	6,10,10b-trihydroxy-3,4a,7,7,10a-pentamethyl-1-oxo-3-vinyldodecahydro-1H-benzo[f]chromen-5-yl acetate	C22H34O7	410.501	-13.97	1.75	86.43	486.04	63	5	0	-6.22	-14.25	1.48	1.72	0.24	-77.42
386	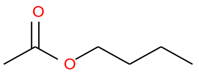	butyl ethanoate	C6H12O2	116.084	-13.96	1.68	21.66	132.52	20	3	-6.52	-3.41	-10.22	3.64	4.52	14.92	-0.53
387	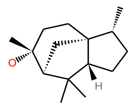	(3R,3aR,6R,7S,8aR)-3,6,8,8-tetramethyloctahydro-1H-3a,7-methanoazulen-6-ol	C15H26O	222.366	-13.93	4.19	16.33	290.46	42	1	-1.28	-4.95	-9.35	0.07	2.65	-0.96	-41.14
388	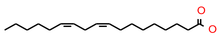	(9Z,12Z)-octadeca-9,12-dienoic acid	C18H32O2	281.248	-13.88	6.73	28.89	353.96	53	15	-2.26	-5.66	-21.71	4.03	6.01	5.8	-51.44
389	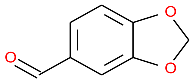	7,9-dioxa-bicyclo[4.3.0]nona-1,3,5-triene-3-carbaldehyde	C8H6O3	150.032	-13.85	1.8	31.51	143.91	17	0	0	-2.68	-14.6	0.07	3.06	0.36	-38.31

390	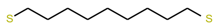	nonane-1,9-dithiol	C9H20S2	192.101	-13.83	4.69	0	198.18	31	10	-2.35	-4.5	-21.93	7.66	9.43	6.04	-98.24
391	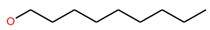	nonan-1-ol	C9H20O	144.151	-13.83	3.52	17.17	175.99	30	8	-3.74	-5.11	-16.57	4.67	8.77	6.44	-86.15
392	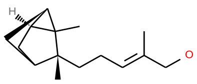	(E)-5-((1R,3R,6S)-2,3-dimethyltricyclo[2.2.1.0 2,6]heptan-3-yl)-2-methylpent-2-en-1-ol	C15H24O	220.35	-13.82	4.6	18.45	302.43	40	5	-2.61	-4.84	-11.26	0	3.29	2.75	-57.2
393	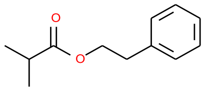	2-phenyl-ethyl 2-methyl-propanoate	C12H16O2	192.115	-13.8	2.88	21.29	200.63	30	4	-2.38	-5.29	-16.82	1.8	8.11	6.22	-98.92
394	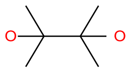	2,3-dimethyl-butane-2,3-diol	C6H14O2	118.099	-13.76	1.03	31.95	150.4	22	3	-2.57	-3.23	-9.02	0.3	4.46	-1.83	-24.68
395	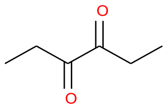	hexane-3,4-dione	C6H10O2	114.068	-13.76	0.55	27	130.25	18	3	-7.05	-2.84	-10.62	0.52	5.13	16.62	-9.85
396	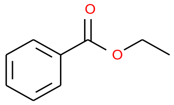	ethyl benzoate	C9H10O2	150.068	-13.74	2.39	20.99	149.49	21	2	0	-4.25	-13.83	0.5	0.66	1.87	-64.68
397	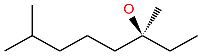	(S)-3,7-dimethyl-octan-3-ol	C10H22O	158.167	-13.74	3.87	16.84	200.41	33	6	-1.12	-5.14	-12.67	1.77	2.9	-0.83	-29.31
398	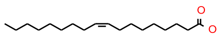	oleic acid	C18H34O2	283.264	-13.7	7.15	28.89	348.59	55	16	-2.59	-6.27	-21.2	6.44	4.78	7.66	-125.83
399	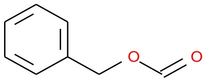	(phenyl-methoxy)-methanone	C8H8O2	136.052	-13.68	1.54	22.57	129.6	18	2	0	-3.18	-13.67	1	2.63	-1.59	-44.47
400	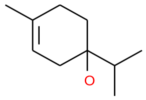	1-isopropyl-4-methyl-cyclohex-3-enol	C10H18O	154.136	-13.66	2.96	15.54	204.53	29	2	-3.3	-4.96	-13.57	2.92	8.73	5.83	-90.16
401	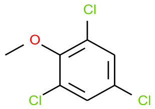	1,3,5-trichloro-2-methoxy-benzene	C7H5Cl3O	209.941	-13.54	3.85	7.96	156.49	16	0	0	-3.86	-12.64	0	1.4	0.96	-28.16
402	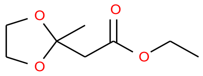	ethyl 2-(2-methyl-1,3-dioxolan-2-yl)-ethanoate	C8H14O4	174.089	-13.5	0.78	36.53	187.9	26	3	0	-4.56	-13.32	0.71	2.58	-1.3	-36.76
403	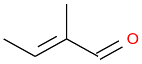	2-methyl-but-2-enal	C5H8O	84.058	-13.47	1.33	14.67	109.37	14	0	0	-2.89	-10.6	0	0.32	-1.78	-21.17
404	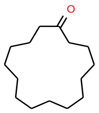	cyclopentadecanone	C15H28O	224.214	-13.46	4.46	14.11	277.17	44	0	0	-5.45	-9.5	0	0.79	-1.36	-64.86

405	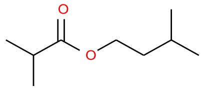	3-methyl-butyl 2-methyl-propanoate	C9H18O2	158.131	-13.41	2.72	21.56	179.73	29	4	-2.86	-5.24	-14.97	2.79	8.6	5.24	-98.04
406	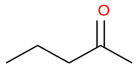	pentan-2-one	C5H10O	86.073	-13.37	1.32	13.84	105.03	16	2	-1.95	-3.22	-11.76	0	3.73	2.26	-62.85
407	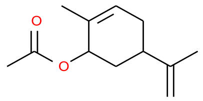	5-isopropenyl-2-methyl-cyclohex-2-enyl ethanoate	C12H18O2	194.131	-13.32	3.23	20.64	245.9	32	2	0	-5.09	-13.44	0	1.36	1.98	-66.94
408	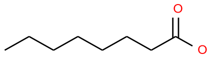	octanoic acid	C8H16O2	144.115	-13.3	2.8	28.89	162.68	25	6	0	-4.06	-9.8	1.8	0.46	-7.17	-34.41
409	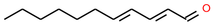	undeca-2,4-dienal	C11H18O	166.136	-13.28	4.02	14.61	221.39	30	5	-2.67	-5.28	-15.85	4.06	8.03	5.83	-92.64
410	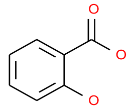	2-hydroxy-benzoic acid	C7H6O3	139.04	-13.27	1.18	45.19	120.18	17	1	-3.56	-2.25	-13.18	0.33	6.35	7.66	-34.13
411	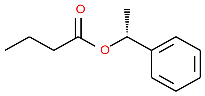	(R)-1-phenyl-ethyl butanoate	C12H16O2	192.115	-13.19	3.09	20.06	203.28	30	4	0	-4.64	-16.26	1.57	1.5	4.02	-70.97
412	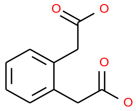	2-(2-(carboxy-methyl)-phenyl)-acetic acid	C10H10O4	194.058	-13.18	0.83	57.01	178.62	22	4	0	-3.66	-12.94	0.72	3.04	-3.74	-31.16
413	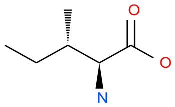	(2S,3S)-2-amino-3-methyl-pentanoic acid	C6H13NO2	132.102	-13.12	-0.45	49.89	132.97	23	5	-6.95	-2.74	-11.66	0	12.95	7.98	-64.46
414	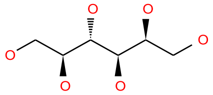	(2S,3R,4S,5S)-hexane-1,2,3,4,5,6-hexaol	C6H14O6	182.079	-13.12	-3.6	98.41	151.83	26	11	-6.1	-2.44	-12.23	2.6	8.85	4.46	-26.88
415	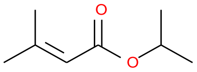	1-methyl-ethyl 3-methyl-but-2-enoate	C8H14O2	142.099	-13.08	2.62	20.35	173.29	24	1	-2.6	-4.93	-13.54	0	8.64	4.95	-90.18
416	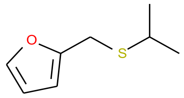	2-((1-methyl-ethylsulfanyl)-methyl)-furan	C8H12OS	156.061	-13.08	2.41	7.99	148.25	22	3	-2.26	-4.86	-15.43	0	7.25	6.17	-87.53
417	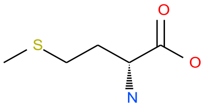	(R)-2-amino-4-methylsulfanyl-butanoic acid	C5H11NO2S	150.059	-13.07	-1.27	49.71	135.27	21	6	-7.53	-3.24	-17.88	2.89	16.7	15	-69.21
418	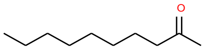	decan-2-one	C10H20O	156.151	-13.07	3.73	13.84	194.56	31	7	0	-5.55	-13.26	2.84	0.46	-0.96	-43.2
419	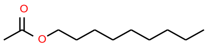	nonyl ethanoate	C11H22O2	186.162	-13.06	4.09	21.66	222.05	35	8	-3.75	-6.02	-15.72	4.14	9.19	6.8	-73.66

420	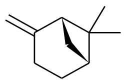	(1R,5R)-6,6-dimethyl-2-methylene-bicyclo[3.1.1]heptane	C10H16	136.125	-13.06	3.72	0	194.46	26	0	0	-3.73	-11.15	0	0	0.9	-29.53
421	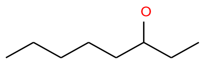	octan-3-ol	C8H18O	130.136	-13.05	3.12	17.13	155.4	27	6	-2.16	-4	-9.93	2.23	2.99	-1.23	-27.83
422	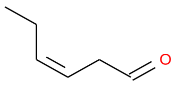	(Z)-hex-3-enal	C6H10O	98.073	-13.03	1.52	14.61	127.43	17	3	0	-3.09	-11.81	0.32	0.37	-1.85	-22.58
423	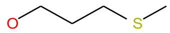	3-methylsulfanyl-propan-1-ol	C4H10OS	106.045	-12.93	0.63	17.17	104.89	16	4	-3.57	-3.31	-12.4	0	7.15	4.51	-59.77
424	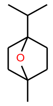	4-isopropyl-1-methyl-7-oxa-bicyclo[2.2.1]heptane	C10H18O	154.136	-12.93	3.06	6.18	205.07	29	1	0	-4.51	-10.78	0.2	0.71	-0.39	-44.34
425	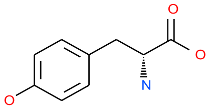	(R)-2-amino-3-(4-hydroxy-phenyl)-propanoic acid	C9H11NO3	182.082	-12.9	-0.57	67.05	162.95	25	5	-8.14	-3.12	-17.94	3.01	19.83	14.94	-84.33
426	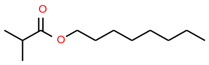	octyl 2-methyl-propanoate	C12H24O2	200.178	-12.87	4.33	21.56	236.13	38	8	-3.28	-6.35	-17.08	3.41	10.18	6.58	-80.25
427	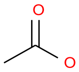	acetic acid	C2H4O2	61.029	-12.87	-0.11	29.33	55.57	9	1	-5.16	-1.41	-7.04	0.03	6.36	4.22	-26.14
428	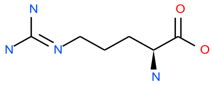	(S)-2-amino-5-((diaminomethylene)amino)pentanoic acid	C6H14N4O2	174.201	-12.86	-2.41	99.14	159.25	27	7	-9.5	-2.21	-20.9	4.53	23.89	17.34	-74.16
429	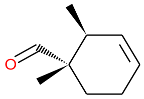	(1S,2S)-1,2-dimethyl-cyclohex-3-enecarbaldehyde	C9H14O	138.104	-12.85	2.12	14.59	179.66	24	1	0	-3.95	-10.09	0.5	0.31	-1.44	-30.63
430	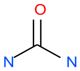	diamino-methanone	CH4N2O	60.032	-12.82	-1.02	55.34	52.76	8	0	-4.96	-0.51	-8.13	0.02	9.46	1.69	-34.19
431	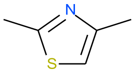	2,4-dimethyl-thiazole	C5H7NS	113.03	-12.8	1.39	9.86	104.79	14	0	0	-3.48	-11.89	0.04	1.51	0.43	-31.82
432	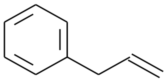	allyl-benzene	C9H10	118.078	-12.79	2.9	0	138	19	2	0	-4.55	-17.76	0.37	5.19	4.43	-78.54
433	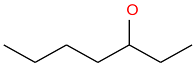	heptan-3-ol	C7H16O	116.12	-12.79	2.64	17.13	137.5	24	5	-2.6	-3.74	-7.87	0.33	2.98	-1.63	-23.44
434	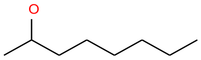	octan-2-ol	C8H18O	130.136	-12.78	3.03	16.99	155.57	27	6	-3.17	-4.82	-14.48	0.72	8.42	4.61	-76.05

435	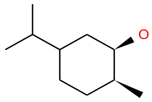	(1R,2S)-5-isopropyl-2-methyl-cyclohexanol	C10H20O	156.151	-12.78	2.95	16.05	179.36	31	2	0	-4.69	-9.85	0	0.09	-1.61	-40.95
436	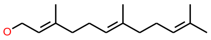	3,7,11-trimethyl-dodeca-2,6,10-trien-1-ol	C15H26O	222.198	-12.77	5.32	17.17	309.64	42	8	-2.98	-6.68	-16.11	0	9.37	5.34	-101.5
437	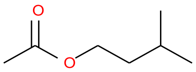	3-methyl-butyl ethanoate	C7H14O2	130.099	-12.71	2	21.66	147.74	23	3	0	-3.89	-12.79	0.67	2.32	-1.3	-39.54
438	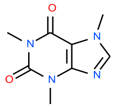	2,4,7-trimethyl-2,4,7,9-tetraaza-bicyclo[4.3.0]nona-1(6),8-diene-3,5-dione	C8H10N4O2	194.08	-12.69	-0.31	43.79	205.73	24	0	-3.92	-3.97	-14.63	0.34	11.45	8.98	-40.88
439	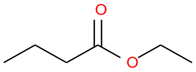	ethyl butanoate	C6H12O2	116.084	-12.68	1.7	20.67	131.06	20	3	0	-4	-11.14	0.97	1.36	-2.55	-25.83
440	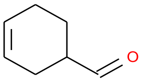	cyclohex-3-enecarbaldehyde	C7H10O	110.073	-12.64	1.67	14.59	140.18	18	1	0	-3.26	-11.12	0	0.74	-0.76	-28.55
441	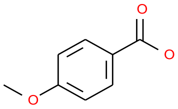	4-methoxy-benzoic acid	C8H8O3	153.055	-12.63	1.65	36.19	140.84	20	1	0	-3.4	-14.96	1.14	4.27	1.22	-33.77
442	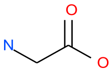	2-amino-acetic acid	C2H5NO2	76.04	-12.61	-1.84	50.55	64.82	11	3	-6.16	-1.09	-9.53	0.11	10.95	4.9	-23
443	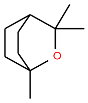	1,3,3-trimethyl-2-oxa-bicyclo[2.2.2]octane	C10H18O	154.136	-12.6	2.93	6.37	202.66	29	0	0	-4.35	-9.73	0.01	1.05	-1.12	-31.03
444	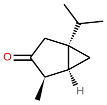	(1R,4R,5S)-1-isopropyl-4-methyl-bicyclo[3.1.0]hexan-3-one	C10H16O	152.12	-12.56	2.44	14.3	195.66	27	1	0	-4.66	-8.98	0.03	0.04	-1.64	-39.08
445	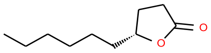	(R)-2-hexyl-5-oxo-tetrahydro-furan	C10H18O2	170.131	-12.52	2.75	21.43	195.53	30	5	0	-5.18	-12.39	0.89	0.72	-0.29	-27.51
446	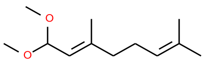	1,1-dimethoxy-3,7-dimethyl-octa-2,6-diene	C12H22O2	198.162	-12.51	3.82	16	253.95	36	6	-3.92	-6.05	-12.24	0	8.7	5.41	-80.5
447	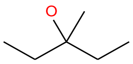	3-methyl-pentan-3-ol	C6H14O	102.104	-12.51	2.1	16.84	131.59	21	3	-1.97	-3.48	-9.55	1.43	2.97	0.5	-23.15
448	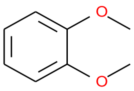	1,2-dimethoxy-benzene	C8H10O2	138.068	-12.47	1.92	15.5	139.09	20	0	-2.26	-4.45	-14.45	8.13	8.5	6.52	-83.58
449	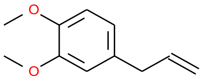	4-allyl-1,2-dimethoxy-benzene	C11H14O2	178.099	-12.43	2.96	15.5	201.27	27	2	-3.3	-5.45	-10.47	0.62	8.61	3.63	-84.84

450	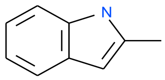	8-methyl-7-aza-bicyclo[4.3.0]nona-1(6),2,4,8-tetraene	C9H9N	131.073	-12.42	2.47	9.39	137.32	19	0	-2.24	-3.9	-10.58	0	4.72	4.4	-74.02
451	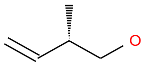	(S)-2-methyl-but-3-en-1-ol	C5H10O	86.073	-12.4	1.05	17.63	103.62	16	3	-1.77	-3.03	-9.76	0.46	3.15	-0.45	-22.34
452	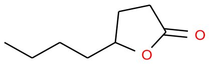	2-butyl-5-oxo-tetrahydro-furan	C8H14O2	142.099	-12.33	1.78	21.43	159.72	24	3	0	-4.05	-10.73	1.2	0.3	-1.38	-37.89
453	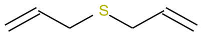	3-allylsulfanyl-prop-1-ene	C6H10S	114.05	-12.27	2.51	0	139.74	17	4	0	-4.31	-13.14	0	0	2.07	-36.42
454	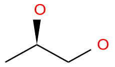	(R)-propane-1,2-diol	C3H8O2	76.052	-12.27	-0.54	34.1	73.97	13	3	-6.01	-1.87	-9.82	1.29	7.84	9.6	-4.51
455	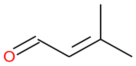	3-methyl-but-2-enal	C5H8O	84.058	-12.25	1.49	14.61	110.87	14	0	0	-2.81	-10.58	0	0.55	-0.31	-32.25
456	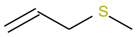	3-methylsulfanyl-prop-1-ene	C4H8S	88.035	-12.23	1.65	0	98.85	13	2	0	-3.76	-13.69	0.16	2.01	1.93	-37.56
457	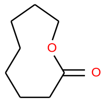	2-oxo-oxonane	C8H14O2	142.099	-12.2	1.14	22.03	163.55	24	0	0	-3.93	-8.98	0	1.18	-2.22	-33.35
458	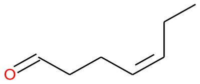	(Z)-hept-4-enal	C7H12O	112.089	-12.14	1.95	14.61	146.82	20	4	0	-3.56	-11.65	0	0.35	-1.17	-26.52
459	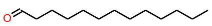	tridecanal	C13H26O	198.198	-12.12	5.31	14.61	247.99	40	11	-2.61	-6.14	-19.11	4.45	9.37	6.19	-75.67
460		methanephosphine	CH5P	48.013	-12.12	0.6	0	25.95	7	1	-2.86	-1.46	-8.59	0.02	4.21	1.4	7.79
461	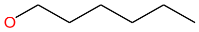	hexan-1-ol	C6H14O	102.104	-12.09	2.08	17.17	122.27	21	5	-2.44	-3.45	-10.05	1.64	3.18	1.62	-47.92
462	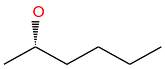	(S)-hexan-2-ol	C6H14O	102.104	-12.08	2.06	16.99	119.76	21	4	-2.13	-3.53	-8.9	2.08	3.1	-0.21	-24.69
463	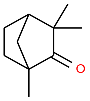	1,3,3-trimethyl-bicyclo[2.2.1]heptan-2-one	C10H16O	152.12	-12.03	2.52	14.23	203.77	27	0	0	-4.08	-10.15	0.04	1.56	-0.49	-44.33
464	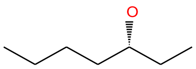	(R)-heptan-3-ol	C7H16O	116.12	-12.02	2.64	17.13	137.5	24	5	-1.37	-4.18	-10.64	1.45	2.8	-0.33	-22.31

465	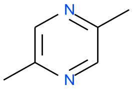	2,5-dimethyl-pyrazine	C6H8N2	108.069	-12	0.52	18.13	109.37	16	0	0	-3.2	-11.39	0	2.2	-0.19	-32.68
466	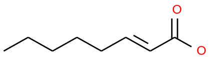	oct-2-enoic acid	C8H14O2	142.099	-11.98	2.94	28.89	169.65	23	5	0	-4.31	-11.56	0.16	2.14	-3.23	-28.03
467	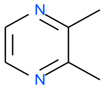	2,3-dimethyl-pyrazine	C6H8N2	108.069	-11.95	0.4	19.49	107.45	16	0	0	-3.49	-10.59	0	1.49	-0.22	-19.01
468	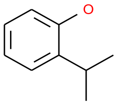	2-isopropyl-phenol	C9H12O	136.089	-11.85	2.6	16.79	141.79	22	1	-2.69	-4.19	-13.43	0.49	8.45	6.43	-76.54
469	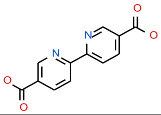	6-(5-carboxy-pyridin-2-yl)-pyridine-3-carboxylic acid	C12H8N2O4	244.048	-11.85	1.19	75.06	206.86	24	2	0	-3.82	-14.79	0.2	6.37	-0.73	-45.47
470	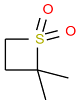	2,2-dimethylthietane 1,1-dioxide	C5H10O2S	134.197	-11.84	1.02	29.29	115.14	18	1	0	-2.37	-10.73	0.05	0	-0.19	-22.66
471	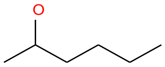	hexan-2-ol	C6H14O	102.104	-11.82	2.06	16.99	119.76	21	4	-1.55	-3.76	-9.72	0.48	2.93	-0.4	-18.75
472	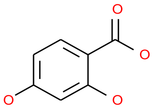	2,4-dihydroxy-benzoic acid	C7H6O4	155.034	-11.73	0.93	62.81	130.8	18	1	-7.74	-2.18	-15.6	3.29	17.17	17.32	-83.65
473	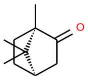	(1R,4R)-1,7,7-trimethyl-bicyclo[2.2.1]heptan-2-one	C10H16O	152.12	-11.73	2.61	14.33	199.91	27	0	0	-4	-8.74	0.06	0.53	-1.09	-35.59
474	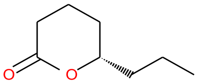	(S)-2-oxo-6-propyl-tetrahydro-2H-pyran	C8H14O2	142.099	-11.73	1.66	21.06	158.82	24	2	0	-4.02	-12	0.23	2.87	-0.58	-31.8
475	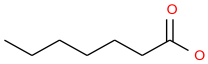	heptanoic acid	C7H14O2	130.099	-11.71	2.32	28.89	144.77	22	5	-6.43	-3.15	1	7.5	5.22	-1.89	-5.36
476	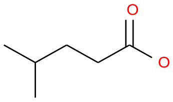	4-methyl-pentanoic acid	C6H12O2	117.092	-11.69	1.67	28.89	124.18	21	4	-2.91	-3.24	-10.93	0.92	4.48	4.57	-20.81
477	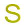	sulfane	H2S	34.0809	-11.61	0.48	0	11.95	3	0	-3.18	-1.89	-5.63	0	4.26	0.19	-4.52
478	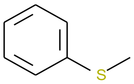	methylsulfanyl-benzene	C7H8S	124.035	-11.36	2.54	0	113.65	16	1	0	-4.3	-16.07	0.17	4.65	5.3	-67.7
479	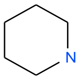	piperidine	C5H11N	85.089	-11.36	0.75	12.39	102.29	17	0	-1.61	-3.05	-7.46	0	2.66	0.34	-18.17

480	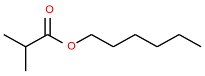	hexyl 2-methyl-propanoate	C10H20O2	172.146	-11.3	3.37	21.56	200.32	32	6	-2.73	-5.6	-14.06	2.6	7.9	5.88	-83.24
481	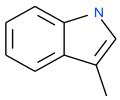	9-methyl-7-aza-bicyclo[4.3.0]nona-1,3,5,8-tetraene	C9H9N	131.073	-11.25	2.48	10.11	135.36	19	0	-1.07	-4.11	-15.29	0	6.73	6.76	-85.68
482	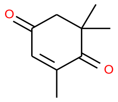	2,6,6-trimethyl-cyclohex-2-ene-1,4-dione	C9H12O2	152.084	-11.22	0.95	26.93	203.88	23	0	0	-3.53	-12.56	0.06	2.01	3.33	-59.9
483	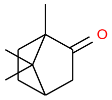	1,7,7-trimethyl-bicyclo[2.2.1]heptan-2-one	C10H16O	152.12	-11.22	2.61	14.33	199.91	27	0	0	-3.92	-8.36	0	0.59	-1.03	-26.62
484	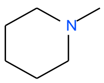	1-methyl-piperidine	C6H13N	99.105	-11.09	1.37	3.91	123.02	20	0	0	-3.52	-10.8	0	0.93	2.08	-40.66
485	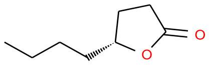	(R)-2-butyl-5-oxo-tetrahydro-furan	C8H14O2	142.099	-11.06	1.78	21.43	159.72	24	3	-1.77	-3.24	-10.52	1.25	3.62	2.42	-32.2
486	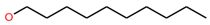	decan-1-ol	C10H22O	158.167	-11.05	4	17.17	193.9	33	9	0	-5.65	-13.62	3.12	1.09	0.22	-41.29
487	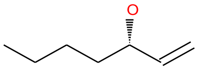	(S)-hept-1-en-3-ol	C7H14O	114.104	-10.94	2.33	17.13	139.88	22	5	0	-3.89	-11.81	0.83	2.44	-2.04	-21.88
488	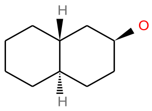	(2S,4aR,8aR)-decahydronaphthalen-2-ol	C10H18O	154.136	-10.92	2.4	16.07	173.04	29	1	0	-4.44	-8.53	0	0.78	-0.9	-45.86
489	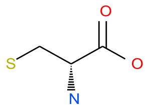	(S)-2-amino-3-mercapto-propanoic acid	C3H7NO2S	122.028	-10.9	-1.75	49.71	96.43	15	5	-8.33	-1.35	-13.91	0.62	17.16	13.82	-44.86
490	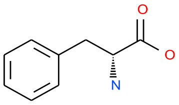	(R)-2-amino-3-phenyl-propanoic acid	C9H11NO2	166.087	-10.86	-0.31	49.44	152.4	24	5	-5.54	-3.51	-18.25	1.2	13.85	15.73	-85.06
491	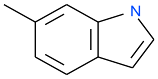	4-methyl-7-aza-bicyclo[4.3.0]nona-1(6),2,4,8-tetraene	C9H9N	131.073	-10.86	2.6	10.02	133.43	19	0	-1.23	-4.21	-15.14	0.02	6.79	7.76	-75.15
492	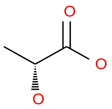	(R)-2-hydroxy-propanoic acid	C3H6O3	91.04	-10.85	-0.71	44.93	79.28	13	3	-5.9	-1.66	-7.37	0	9.14	5.91	-38.03
493	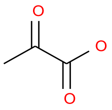	2-oxo-propanoic acid	C3H4O3	89.024	-10.84	-0.49	42.74	81.86	11	2	-4.67	-1.46	-10.83	0	8.59	7.8	-26.96
494		methanethiol	CH4S	48.003	-10.82	0.89	0	32.86	6	1	-2.49	-1.41	-7.83	0.03	2.5	2.68	-3.35

495	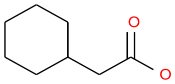	2-cyclohexyl-acetic acid	$C_8H_{14}O_2$	143.107	-10.81	2.07	28.81	153.55	25	3	-2.24	-3.67	-10.97	0.54	3.96	5.3	-26.73
496	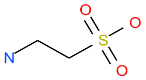	2-amino-ethanesulfonic acid	$C_2H_7NO_3S$	125.015	-10.67	-2.88	64.72	88.88	14	4	-8.32	-1.18	-9.09	1.08	9.59	16.17	-5.29
497	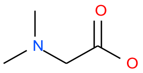	2-dimethylamino-acetic acid	$C_4H_9NO_2$	104.071	-10.6	-0.76	32.78	106.75	17	3	-4.86	-2.6	-7.02	0.43	8.19	3.79	-37.99
498	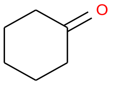	cyclohexanone	$C_6H_{10}O$	98.073	-10.47	1.24	13.78	116.59	17	0	0	-3.2	-9.18	0	0.4	0.83	-40.77
499	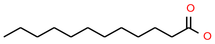	dodecanoic acid	$C_{12}H_{24}O_2$	200.178	-10.45	4.73	28.89	234.3	37	10	-1.71	-5.91	-16.7	3.66	9.86	1.75	-79.91
500	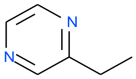	2-ethyl-pyrazine	$C_6H_8N_2$	108.069	-10.39	0.64	19.3	106.74	16	1	-6.77	-3.01	-8.01	0.95	4.79	19.05	5.76
501	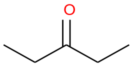	pentan-3-one	$C_5H_{10}O$	86.073	-10.38	1.34	13.27	106.68	16	2	0	-3.07	-10.1	0.05	1.23	-0.52	-23.64
502	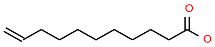	undec-10-enoic acid	$C_{11}H_{20}O_2$	185.154	-10.29	3.86	28.89	220.24	34	10	-3.94	-5.35	-22.81	5.25	12.64	16.04	-117.71
503	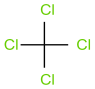	tetrachloro-methane	CCl_4	151.875	-10.26	2.65	0	88.64	5	0	0	-3.27	-8.31	0	0	0.37	-8.63
504	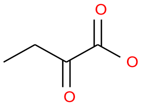	2-oxo-butanoic acid	$C_4H_6O_3$	103.04	-10.22	0	42.17	99.08	14	3	-4.68	-1.98	-10.66	0	8.5	8.22	-28.88
505	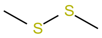	(methyldisulfanyl)methane	$C_2H_6S_2$	94.199	-10.18	1.31	0	63.33	10	1	0	-3.2	-8.02	0.12	0	-0.94	-7.32
506	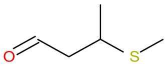	3-methylsulfanyl-butanal	$C_5H_{10}OS$	118.045	-10.15	0.8	14.61	115.45	17	3	0	-3.29	-11.03	0	1.21	0.56	-24.55
507	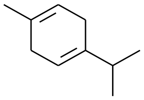	1-isopropyl-4-methyl-cyclohexa-1,4-diene	$C_{10}H_{16}$	136.125	-10.07	4.57	0	191.39	26	1	0	-5.08	-13.66	0	5.07	3.92	-84.49
508	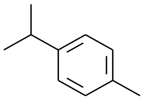	p-cymene	$C_{10}H_{14}$	134.11	-9.99	3.38	0	149.9	24	1	0	-4.95	-13.67	2.11	4.64	4.43	-93.48
509	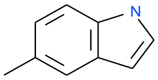	3-methyl-7-aza-bicyclo[4.3.0]nona-1(6),2,4,8-tetraene	C_9H_9N	131.073	-9.99	2.6	10.02	133.43	19	0	-0.79	-4.1	-15.36	0	6.48	7.97	-86.23

510	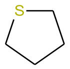	tetrahydro-thiophene	C4H8S	88.035	-9.91	1.74	0	89.52	13	0	0	-3.15	-8.79	0	0	1.5	-34.39
511	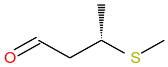	(S)-3-methylsulfanyl-butanal	C5H10OS	118.045	-9.86	0.8	14.61	115.45	17	3	0	-3.43	-10.37	0	0.64	0.82	-35.38
512	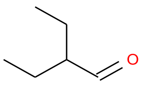	2-ethyl-butanal	C6H12O	100.089	-9.84	1.87	14.67	119.58	19	3	0	-3.44	-8.36	0.09	0.44	-1.99	-29.33
513	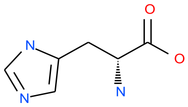	(R)-2-amino-3-(3H-imidazol-4-yl)-propanoic acid	C6H9N3O2	156.077	-9.63	-2.32	71.23	137.25	21	5	-2.71	-2.53	-16.2	1.5	8.3	8.94	-49.08
514	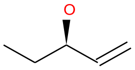	(R)-pent-1-en-3-ol	C5H10O	86.073	-9.55	1.36	17.13	104.12	16	3	-5.31	-2.94	-11.74	3.24	4.86	18.4	-4.97
515	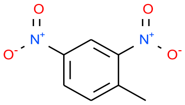	1-methyl-2,4-dinitro-benzene	C7H6N2O4	182.033	-9.5	1.59	76.46	147.87	19	2	0	-2.94	-17.34	0	5.38	6.93	-74.23
516	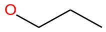	propan-1-ol	C3H8O	60.058	-9.5	0.63	17.17	68.56	12	2	-6.16	-2.23	-9.53	1.1	4.84	18.62	3.35
517	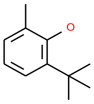	2-methyl-6-tert-butyl-phenol	C11H16O	164.12	-9.49	3.37	15.72	191.79	28	1	-2.63	-4.96	-11.93	1.31	8.69	8.04	-99
518		dodecan-1-ol	C12H26O	186.198	-9.49	4.97	17.17	229.71	39	11	-3.61	-6.34	-14.03	4.58	10.32	5.41	-70.22
519	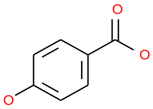	4-hydroxy-benzoic acid	C7H6O3	139.04	-9.49	1.3	46.26	119.55	17	1	-2.91	-2.27	-14.6	0.43	7.98	11.22	-38.92
520	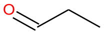	propionaldehyde	C3H6O	58.042	-9.44	0.49	14.61	68.34	10	1	-3.71	-1.7	-12.83	0.17	3.35	16.39	-9.77
521	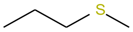	1-methylsulfanyl-propane	C4H10S	90.05	-9.42	1.79	0	96.91	15	2	0	-3.77	-11.78	0	2.12	3.18	-53.07
522	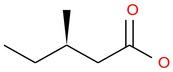	(R)-3-methyl-pentanoic acid	C6H12O2	117.092	-9.4	1.77	28.89	123.04	21	4	-4.28	-3.1	-14.12	1.34	10.34	11.3	-75.97
523	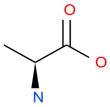	(S)-2-amino-propanoic acid	C3H7NO2	90.056	-9.38	-1.85	49.27	84.13	14	3	-6.05	-1.67	-12.93	0.34	12.15	13.72	-48.86
524	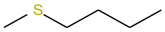	1-methylsulfanyl-butane	C5H12S	104.066	-9.35	2.27	0	114.81	18	3	0	-4.19	-11.78	0	2.17	2.74	-50.3

525	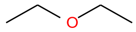	ethoxy-ethane	C4H10O	74.073	-9.35	1.13	8.19	87.88	15	2	0	-3.16	-8.07	0.18	1	-1.68	-17.51
526	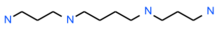	N1,N4-di(3-amino-propyl)-butane-1,4-diamine	C10H26N4	202.216	-9.25	-1.5	66.26	232.67	40	13	-6.86	-5.43	-17.15	3.32	19.89	9.35	-51.92
527	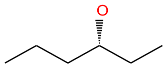	(R)-hexan-3-ol	C6H14O	102.104	-8.99	2.16	17.13	119.59	21	4	0	-3.96	-8.27	1.84	0.61	-1.41	-32.55
528	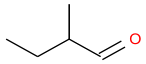	2-methyl-butanal	C5H10O	86.073	-8.89	1.29	14.67	99.41	16	2	0	-3.08	-7.13	0.03	0.29	-1.69	-23.97
529	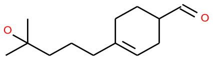	4-(4-hydroxy-4-methyl-pentyl)-cyclohex-3-enecarbaldehyde	C13H22O2	210.162	-8.86	3.16	30.78	265.44	37	6	-4.78	-5.35	-11.14	1.51	11.13	9.09	-47.92
530	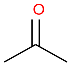	propan-2-one	C3H6O	58.042	-8.78	0.35	14.42	68.93	10	0	-3.24	-2.13	-12.65	0	3.41	16.58	-7.64
531	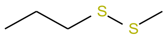	1-(methyldisulfanyl)propane	C4H10S2	122.252	-8.75	2.32	0	107.24	16	3	0	-4.38	-15.11	0.21	5.28	5.34	-54.72
532	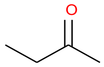	butan-2-one	C4H8O	72.058	-8.69	0.84	13.84	87.57	13	1	-3.1	-2.61	-12.88	0	3.42	16.09	-14.89
533	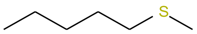	1-methylsulfanyl-pentane	C6H14S	118.082	-8.6	2.76	0	132.72	21	4	0	-4.74	-15.05	0.95	6.02	4.12	-69.63
534	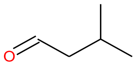	3-methyl-butanal	C5H10O	86.073	-8.34	1.3	14.61	100.67	16	2	0	-2.9	-7.8	0.16	0.43	-0.18	-16.71
535	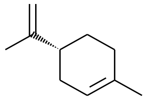	(R)-4-isopropenyl-1-methyl-cyclohex-1-ene	C10H16	136.125	-8.27	3.83	0	195.06	26	1	0	-5.28	-14.84	0	6.07	7.5	-79.37
536	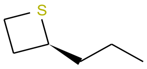	(S)-2-propyl-thietane	C6H12S	116.066	-7.92	3.16	0	130.89	19	2	0	-4.11	-10.85	0	3.61	2.68	-59.51
537		hexadecan-1-ol	C16H34O	242.261	-7.85	6.9	17.17	301.34	51	15	0	-6.57	-14.98	5.03	3.4	-0.03	-68.43
538	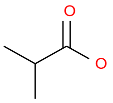	isobutyric acid	C4H8O2	89.06	-7.62	0.61	28.92	88.91	15	2	-5.54	-2.44	-10.96	0	9.89	15.73	-56.17
539	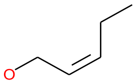	(Z)-pent-2-en-1-ol	C5H10O	86.073	-7.3	1.3	17.17	108.87	16	3	-5.31	-2.9	-6.63	0	4.96	13.93	-9.59

540	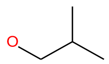	2-methyl-propan-1-ol	C4H10O	74.073	-7.28	0.95	17.63	83.72	15	2	-6.24	-2.62	-7.04	1.41	4.92	18.82	-10.99
541	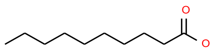	decanoic acid	C10H20O2	173.154	-7.26	3.76	28.89	198.49	33	9	-2.06	-4.92	-23.57	4.71	12.43	15.3	-118.83
542	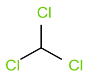	chloroform	CHCl3	117.914	-7.16	1.84	0	63.07	5	0	0	-3.11	-6.37	0	0.87	0.96	6.05
543	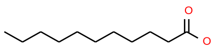	undecanoic acid	C11H22O2	187.17	-6.89	4.24	28.89	216.39	36	10	-3.2	-5.29	-21.16	7.49	12.57	15.91	-111.19
544	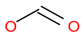	formic acid	CH2O2	47.013	-6.82	-0.34	29.25	36.86	6	1	-3.93	-0.33	-6.38	0.01	6.95	5.89	-20.27
545	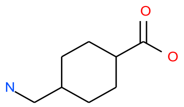	4-(amino-methyl)-cyclohexanecarboxylic acid	C8H15NO2	158.118	-6.74	0.13	50.5	163.05	27	4	-7.01	-3.3	-12.3	0.86	16.91	15.79	-87.68
546	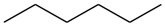	hexane	C6H14	86.11	-6.7	3.24	0	114.29	20	3	0	-4.08	-10.96	0.04	4.06	3.26	-60.93
547	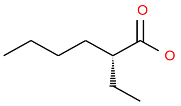	(R)-2-ethyl-hexanoic acid	C8H16O2	145.123	-6.56	2.73	28.92	160.14	27	6	-5.48	-4.03	-14.05	7.08	11.91	17.44	-78.5
548	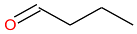	butyraldehyde	C4H8O	72.058	-6.45	0.98	14.61	86.83	13	2	-4.2	-2.1	-8.11	0.93	3.35	15.12	1.78
549	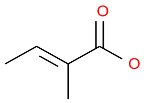	2-methyl-but-2-enoic acid	C5H8O2	101.06	-6.17	1.24	28.92	118.77	16	1	-3.69	-2.95	-13.73	0	11.85	14.21	-49.39
550	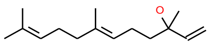	3,7,11-trimethyl-dodeca-1,6,10-trien-3-ol	C15H26O	222.198	-6.15	5.24	16.84	317.23	42	8	-2.19	-6.46	-16.29	0	11.83	9.61	-71.33
551	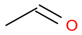	acetaldehyde	C2H4O	44.026	-5.26	0.09	14.15	48.44	7	0	-3.22	-1.25	-9.97	0	3.23	17.08	-6.04
552	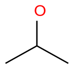	propan-2-ol	C3H8O	60.058	-5.05	0.52	16.85	65.31	12	1	-3.98	-2.32	-8.83	0.06	4.86	16.9	-8.88
553	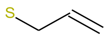	prop-2-ene-1-thiol	C3H6S	74.019	-5.04	1.65	0	77.56	10	2	-1.55	-2.1	-14.39	0	3.35	16.63	-16.28
554	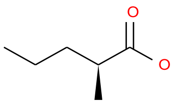	(S)-2-methyl-pentanoic acid	C6H12O2	117.092	-4.98	1.67	28.92	124.09	21	4	-4.02	-3.42	-15.71	0.93	11.56	18.38	-72.32

555	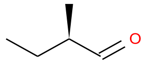	(R)-2-methyl-butanal	C5H10O	86.073	-4.88	1.29	14.67	99.41	16	2	-3.49	-2.55	-8.68	1.25	3.42	16.03	-12.12
556	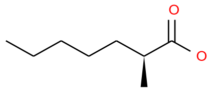	(S)-2-methyl-heptanoic acid	C8H16O2	145.123	-4.67	2.63	28.92	159.9	27	6	-3.79	-4.09	-16.92	4.38	12.23	17.89	-83.12
557	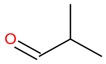	isobutyraldehyde	C4H8O	72.058	-4.54	0.72	14.67	82.56	13	1	-3.45	-2.13	-10.65	0.17	3.45	19.83	-10.99
558	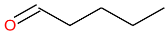	pentanal	C5H10O	86.073	-4.48	1.46	14.61	104.74	16	3	-4.54	-2.5	-5.96	2.72	3.48	15.5	-4.73
559	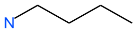	butan-1-amine	C4H11N	73.089	-4.45	0.51	21.67	88.53	16	3	-3.98	-2.6	-8.99	1.26	6.36	14.82	-6.14
560	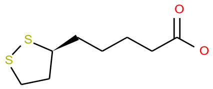	(S)-5-(1,2-dithiolan-3-yl)-pentanoic acid	C8H14O2S2	207.051	-3.51	2.96	29.18	187.75	27	6	-2.24	-4.67	-16.9	1.21	11.98	14.54	-98.17
561	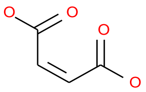	maleic acid	C4H4O4	118.027	-3.29	0.23	57.28	110.73	14	2	-4.82	-1.54	-14.72	2.13	14.44	19.15	-43.55
562	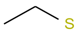	ethanethiol	C2H6S	62.019	-2.76	1.31	0	58.66	9	1	-1.45	-1.71	-10.2	0.23	3.14	14.08	6.22
563	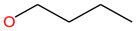	butan-1-ol	C4H10O	74.073	-2.2	1.11	17.17	86.46	15	3	-3.77	-2.63	-7	1.49	4.96	16.04	-12.8
564	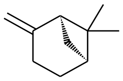	(1S,5S)-6,6-dimethyl-2-methylene-bicyclo[3.1.1]heptane	C10H16	136.125	-2.17	3.72	0	194.46	26	0	0	-4.77	-7.01	0.42	5.73	5.65	-74.76
565	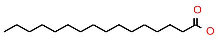	palmitic acid	C16H32O2	257.248	-1.39	6.65	28.89	305.93	51	15	-1.53	-7.58	-19.36	8.36	11.45	14.13	-97.38
566	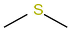	methylsulfanyl-methane	C2H6S	62.019	-0.83	0.79	0	54.94	9	0	0	-2.79	-9.47	0.06	1.69	14.01	-2.49
567	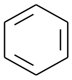	benzene	C6H6	78.047	-0.76	1.86	0	75.9	12	0	0	-2.9	-11.06	0	1.5	16.85	5.49
568	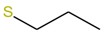	propane-1-thiol	C3H8S	76.035	-0.68	1.8	0	75.66	12	2	-1.33	-2.12	-9.25	1.04	3.34	14.62	-10.13
569	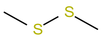	dimethyl disulphide	C2H6S2	93.991	1.01	1.31	0	63.33	10	1	0	-3.22	-10.1	0.13	1.69	17.27	-9.43

570	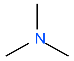	N,N-dimethyl-methanamine	C3H9N	59.073	1.22	0.14	3.81	76.7	13	0	0	-2.82	-9.58	0.11	2.88	15.96	-2.27
571	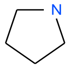	pyrrolidine	C4H9N	71.073	1.24	0.39	12.76	85.28	14	0	-2.64	-2.56	-4.24	0	4.61	15.77	10.62
572	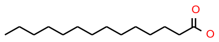	tetradecanoic acid	C14H28O2	229.217	1.74	5.69	28.89	270.11	45	13	-3.37	-6.7	-14.58	6.77	13.38	17.42	-109.77
573	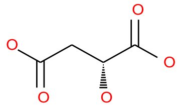	(R)-2-hydroxy-succinic acid	C4H6O5	136.037	2.82	-1.54	73.45	108.21	17	6	-8.7	-1.4	-15.43	0.89	20.92	33.19	-74.16
574	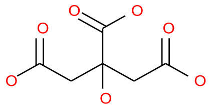	2-hydroxy-propane-1,2,3-tricarboxylic acid	C6H8O7	195.05	20.17	-1.97	101.69	167.15	24	9	-6.81	-1.63	-18.69	0.75	23.43	51.85	-84.66
